# Tumors produce glucocorticoids by metabolite recycling, not synthesis, and activate Tregs to promote growth

**DOI:** 10.1172/JCI164599

**Published:** 2023-09-15

**Authors:** Matthew D. Taves, Shizuka Otsuka, Michaela A. Taylor, Kaitlynn M. Donahue, Thomas J. Meyer, Margaret C. Cam, Jonathan D. Ashwell

**Affiliations:** 1 Laboratory of Immune Cell Biology and; 2 CCR Collaborative Bioinformatics Resource, Center for Cancer Research, National Cancer Institute (NCI), NIH, Bethesda, Maryland, USA.

**Keywords:** Immunology, Oncology, Adaptive immunity, Cancer

## Abstract

Glucocorticoids are steroid hormones with potent immunosuppressive properties. Their primary source is the adrenals, where they are generated via de novo synthesis from cholesterol. In addition, many tissues have a recycling pathway in which glucocorticoids are regenerated from inactive metabolites by the enzyme 11β-hydroxysteroid dehydrogenase type 1 (11β-HSD1, encoded by *Hsd11b1*). Here, we find that multiple tumor types express *Hsd11b1* and produce active glucocorticoids. Genetic ablation of *Hsd11b1* in such cells had no effect on in vitro growth, but reduced in vivo tumor progression, which corresponded with increased frequencies of CD8^+^ tumor-infiltrating lymphocytes (TILs) expressing activation markers and producing effector cytokines. Tumor-derived glucocorticoids were found to promote signatures of Treg activation and suppress signatures of conventional T cell activation in tumor-infiltrating Tregs. Indeed, CD8^+^ T cell activation was restored and tumor growth reduced in mice with Treg-specific glucocorticoid receptor deficiency. Importantly, pharmacologic inhibition of 11β-HSD1 reduced tumor growth to the same degree as gene knockout and rendered immunotherapy-resistant tumors susceptible to PD-1 blockade. Given that *HSD11B1* expression is upregulated in many human tumors and that inhibition of 11β-HSD1 is well tolerated in clinical studies, these data suggest that targeting 11β-HSD1 may be a beneficial adjunct in cancer therapy.

## Graphical abstract

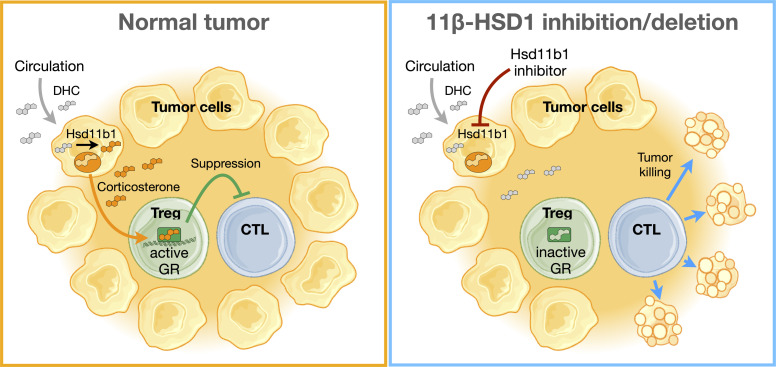

## Introduction

Glucocorticoids are adrenal-derived hormones that circulate in the blood to maintain systemic homeostasis and coordinate responses to changing environmental conditions and stressors (1). Glucocorticoids exert pleiotropic actions by binding the ubiquitously expressed glucocorticoid receptor (GR) and have especially pronounced effects on metabolism and immunity. Systemic glucocorticoids are potently immunosuppressive and are essential to preventing runaway immune responses (2). Indeed, although promoting pathogen clearance, loss of adrenal glucocorticoid production increases mortality due to vascular shock and unrestrained inflammation. Glucocorticoid immunosuppression is particularly pronounced in T cells, and specific ablation of T cell GR in mice infected with *Toxoplasma gondii* results in massive cytokine production and increased mortality (3), similar to that seen with loss of circulating glucocorticoids. Glucocorticoids can act at multiple levels to suppress T cell effector responses: inhibition of dendritic cell antigen presentation and cytokine production, inhibition of helper T cell differentiation, and exacerbation of T cell dysfunction (4). Additionally, recent findings suggest that immunosuppression caused by exogenous glucocorticoid administration can also be largely due to their stimulatory effects on Tregs (5, 6). In this way, glucocorticoids act on T cells by a variety of mechanisms to control the immune response.

Glucocorticoids are synthesized de novo in the adrenals by sequential modification of cholesterol by mitochondrial and microsomal enzymes. The final step in this biosynthetic pathway is catalyzed by P450c11β (CYP11B1, encoded by *Cyp11b1*), a cytochrome P450 enzyme that converts steroid precursors into active glucocorticoids, cortisol in humans, and corticosterone in mice. In kidney and colon cells, these active glucocorticoids are metabolized (inactivated) by the enzyme 11β-hydroxysteroid dehydrogenase type 2 (11β-HSD2, encoded by *Hsd11b2*), producing cortisone (human) or dehydrocorticosterone (mouse). These inactive species are present at high levels in the blood (7, 8) and can be converted back to their active counterparts by 11β-HSD1 (11β-hydroxysteroid dehydrogenase type 1, encoded by *Hsd11b1*), providing a mechanism of glucocorticoid generation distinct from de novo synthesis. 11β-HSD1 expression is highest in the liver, but it is also expressed in many other tissues, including adipose tissues, brain, and lymphoid organs (9, 10).

Although adrenal-derived glucocorticoids are a central and critical mediator of homeostatic responses, other tissues also express the machinery needed to produce glucocorticoids de novo. This is particularly the case at sites of immune cell activation such as the thymus (11–13), intestine (14, 15), and skin (16–18). As with systemic glucocorticoids, paracrine glucocorticoid production functions to limit immune activation, but in a way that avoids the detrimental effects of systemic glucocorticoid exposure. Extra-adrenal glucocorticoid production may allow effective local immunosuppression and occurs at locations of massive cell proliferation and high mutational burden (19). This raises the possibility that the glucocorticoid synthetic machinery could be coopted by tumor cells to escape immune detection or destruction. Recent reports have proposed that de novo glucocorticoid production by infiltrating hematopoietic cells within the tumor microenvironment in fact promotes tumor growth in vivo (20, 21). Both *CYP11B1* transcripts and 11β-HSD1 protein have been detected in primary human tumors (22, 23), and cultured colorectal tumor tissue can produce cortisol (23). We aimed to determine, therefore, whether active glucocorticoids are produced by tumor cells themselves and, if so, determine their biosynthetic pathway and biological significance.

## Results

### Cancer cells generate glucocorticoids by recycling inactive metabolites.

**Figure 1:**
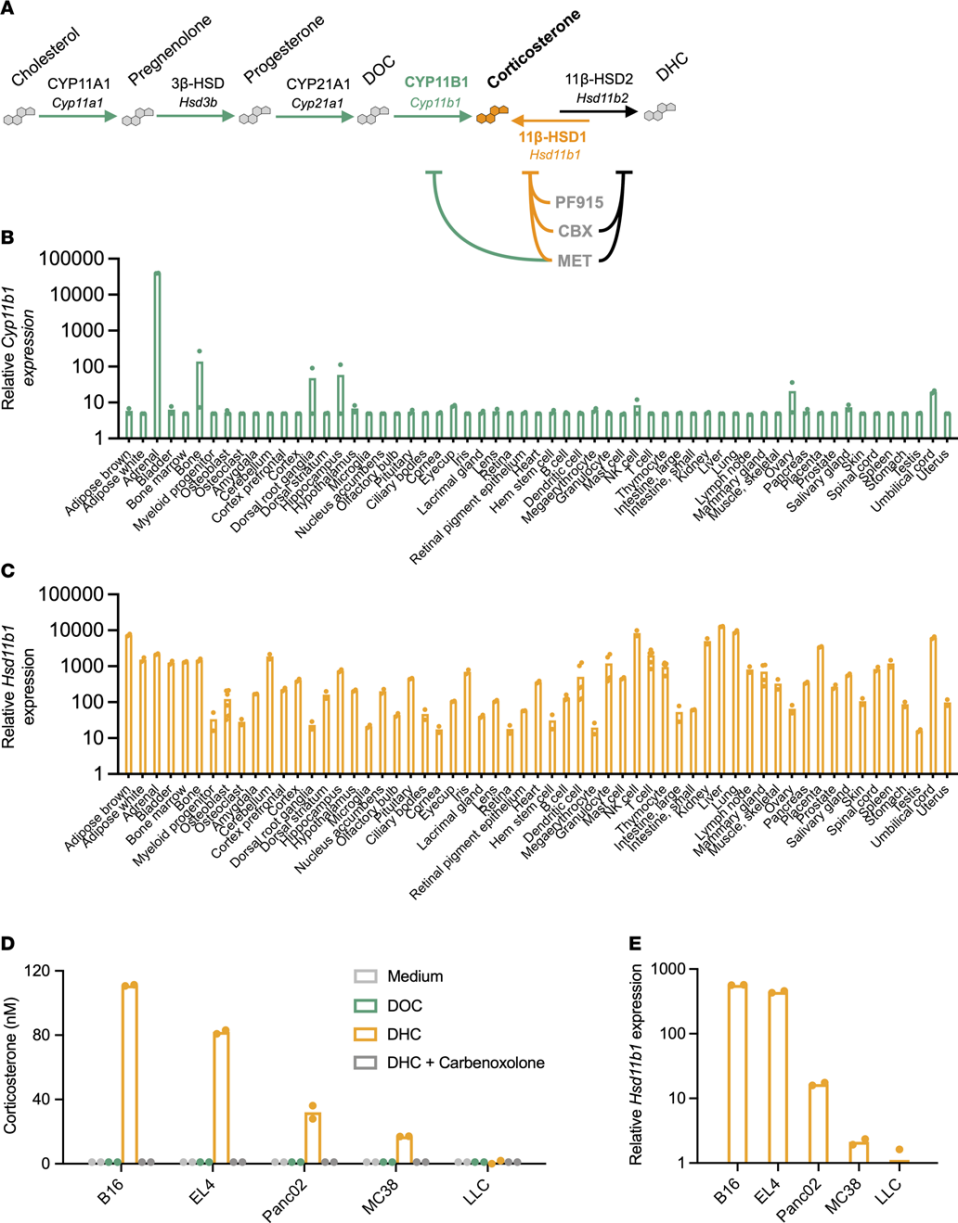
Widespread glucocorticoid generation via 11β-HSD1 activity in healthy tissues and tumor cells. (**A**) Simplified glucocorticoid synthetic pathway. Green arrows indicate enzyme-mediated steroid conversions required for de novo corticosterone biosynthesis from cholesterol, including corticosterone generation from its immediate precursor DOC by CYP11B1 (or P450c11β, encoded by *Cyp11b1*), orange indicates corticosterone generation from inactive metabolite DHC by 11β-HSD1 (*Hsd11b1*), and black indicates corticosterone inactivation to DHC by 11β-HSD2 (*Hsd11b2*). Pharmacological inhibitors are listed in order of decreasing specificity (PF-915275 [PF915]; carbenoxolone [CBX]; metyrapone [MET]) and are indicated along with their target enzymes. (**B** and **C**) Relative gene expression of *Cyp11b1* and *Hsd11b1* in adult mouse tissues. Data were acquired from BioGPS, and bars show the means of 2 or more samples per tissue. (**D**) Corticosterone production by mouse tumor cell lines. 5 × 10^4^ B16 melanoma, EL4 lymphoma, Panc02 pancreatic adenocarcinoma, MC38 colon carcinoma, and Lewis lung carcinoma cells were cultured for 45 minutes with 100 μM metyrapone or carbenoxolone and then cultured overnight with 100 nM DOC or DHC to test CYP11B1 or 11β-HSD1 activity, respectively. Supernatants were diluted in assay buffer and quantified via ELISA. (**E**) *Hsd11b1* gene expression by mouse tumor cell lines. Total RNA was extracted from tumor cell lines and relative *Hsd11b1* gene expression quantified via RT-qPCR, normalized to *18S* RNA expression. **D** and **E** show the means of duplicate wells from 1 of 2 independent experiments. Supporting data are available in Supplemental Figure 1.

Active glucocorticoids are synthesized de novo from cholesterol via activity of the cholesterol transporter StAR and sequential metabolic activities of CYP11A1 (*Cyp11a1*), 3β-HSD (*Hsd3b*), CYP21A1 (*Cyp21*), and CYP11B1 (*Cyp11b1*). A second pathway allows active glucocorticoids to be regenerated from inactive metabolites via the activity of 11β-HSD1 (Figure 1A and Supplemental Figure 1A; supplemental material available online with this article; https://doi.org/10.1172/JCI164599DS1). BioGPS analysis of global gene expression data from healthy adult mice (24) showed that *Cyp11b1* expression is high in the adrenals, as expected, but quite low in nearly all other tissues (Figure 1B and Supplemental Figure 1B). In contrast, *Hsd11b1* is highly expressed in liver as well as the majority of other tissues examined (Figure 1C and Supplemental Figure 1B). Thus, 11β-HSD1 could possibly be a widespread source of tissue glucocorticoid amplification.

Mouse tumor cell lines were tested for the presence of functional glucocorticoid-synthetic machinery. Cells cultured with the immediate precursor of corticosterone, 11-deoxycorticosterone (DOC), did not produce corticosterone, indicating the absence of constitutive CYP11B1 activity (Figure 1D). To see whether CYP11B1 activity was inducible by inflammatory mediators, as it is in skin (16) and intestine (15), B16 (melanoma) or MC38 (colon carcinoma) cells were stimulated with cAMP, phorbol 12-myristate 13-acetate (PMA), LPS, TNF-α, or IL-6; all failed to induce CYP11B1 conversion of DOC to corticosterone (Supplemental Figure 1, C and D). These results indicate that, even in the presence of inflammatory signals, these tumors have little capacity for de novo glucocorticoid synthesis. In contrast, when cultured with the inactive glucocorticoid metabolite 11-dehydrocorticosterone (DHC) (equivalent to cortisone in humans), 4 of the 5 tumor lines were able to produce corticosterone (Figure 1D). B16 melanoma cells in particular converted all of the available DHC substrate into active corticosterone, with progressively less production by EL4 (thymoma), Panc02 (pancreatic adenocarcinoma), MC38 (colon carcinoma), and LLC (Lewis lung carcinoma). Corticosterone regeneration from DHC was completely blocked by the 11β-HSD inhibitor carbenoxolone, demonstrating that corticosterone was indeed produced by the glucocorticoid “recycling” pathway. Differences in 11β-HSD1 activity paralleled *Hsd11b1* gene expression (Figure 1E), and *Hsd11b1* gene expression was much higher than *Cyp11b1* expression in all glucocorticoid-producing cell lines (Supplemental Figure 1E). Expression of *Hsd11b1* was also higher than that of *Hsd11b2*, which encodes the isoenzyme 11β-HSD2, which instead converts active glucocorticoids to their inactive metabolites. The single exception was LLC cells, which produced no glucocorticoids, possibly due to higher expression of *Hsd11b2* than *Hsd11b1* (Supplemental Figure 1E). This was furthermore reflected in the relative *Hsd11b1*/*Hsd11b2* expression patterns across healthy tissues (Supplemental Figure 1B). Thus, 11β-HSD1 is widely expressed and active in a range of cancer cells.

### 11β-HSD1 activity promotes tumor growth in vivo but not in vitro.

**Figure 2:**
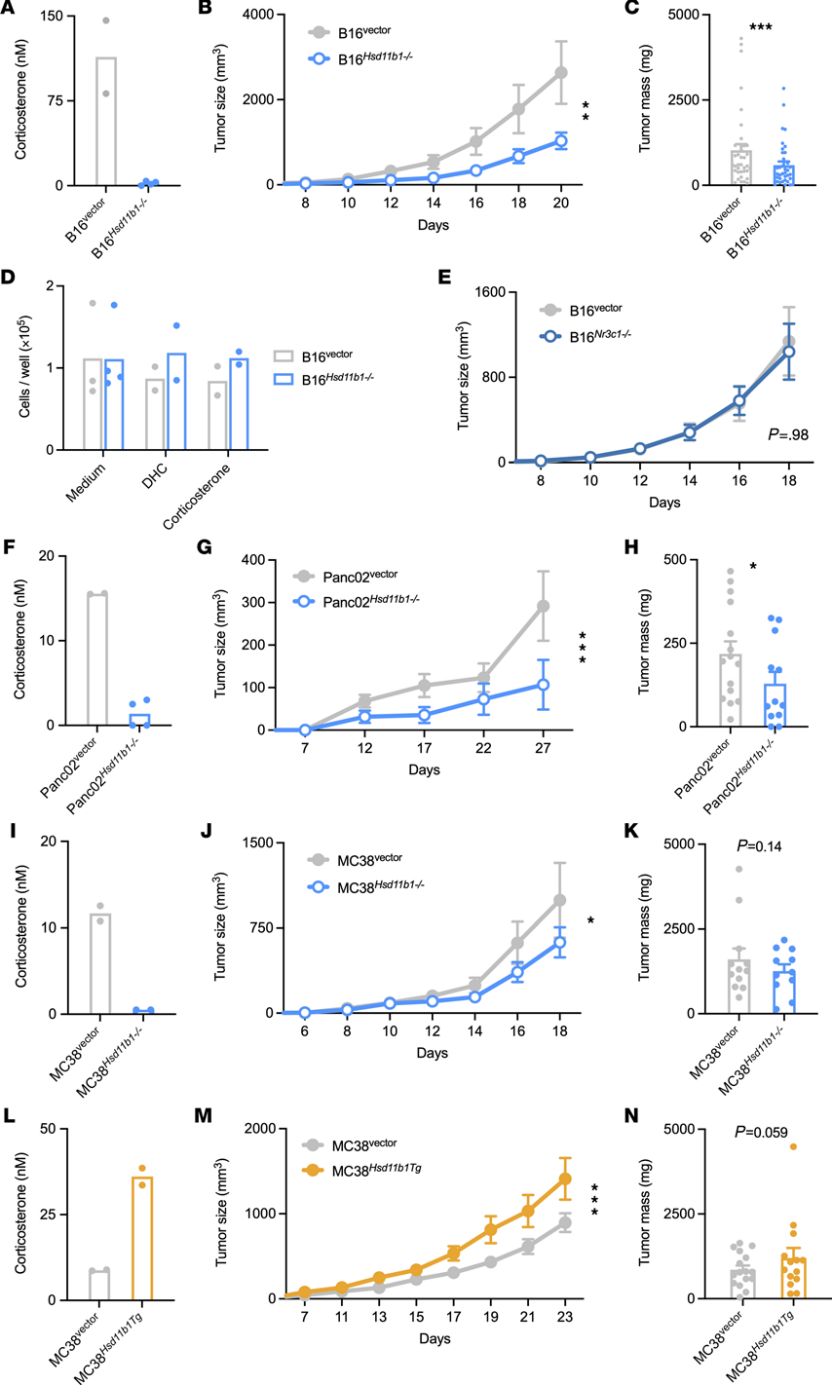
11β-HSD1 expression by cancer cells promotes tumor growth in vivo. (**A**) B16 control and *Hsd11b1^–/–^* cell corticosterone generation in vitro after overnight incubation with 100 nM DHC. Representative of 2 independent experiments. (**B**) B16 control and *Hsd11b1^–/–^* cell tumor growth in WT mice (*n* = 8, 9). Representative of 7 experiments. (**C**) B16 control and *Hsd11b1^–/–^* cell tumor masses (*n* = 37, 37). Pooled from 7 experiments. (**D**) B16 control and *Hsd11b1^–/–^* cell growth in vitro after 72 hours in standard growth medium or supplemented with 100 nM DHC or corticosterone. (**E**) B16 control and *Nr3c1^–/–^* cell tumor growth in WT mice (*n* = 11, 12). Representative of 2 experiments. (**F**–**H**) Panc02 control and *Hsd11b1^–/–^* cell corticosterone generation in vitro, tumor growth (*n* = 8, 6; representative of 2 experiments), and tumor masses (*n* = 15, 12; pooled from 2 experiments). (**I**–**K**) MC38 control and *Hsd11b1^–/–^* cell corticosterone generation in vitro, tumor growth (*n* = 6, 7; representative of 2 experiments), and tumor masses (*n* = 12, 11; pooled from 2 experiments). (**L**–**N**) MC38 control and *Hsd11b1^Tg^* cell corticosterone generation in vitro, tumor growth (*n* = 8, 6; representative of 2 experiments), and tumor masses (*n* = 16, 14; pooled from 2 experiments). Tumor growth was analyzed using repeated-measures ANOVA (rmANOVA) with cell genotype and mouse sex as factors. Tumor mass was analyzed using ANOVA with cell genotype, mouse sex, and experiment as factors. Tumor data are represented as means ± SEM. *P < 0.05;**P < 0.01; ***P < 0.001. Supporting data are available in Supplemental Figure 2.

To determine whether tumor cell–generated glucocorticoids have an effect on tumor growth, CRISPR/Cas9 targeting was used to generate control and *Hsd11b1*-deficient tumor cell lines. B16 *Hsd11b1^–/–^* cells were unable to regenerate corticosterone in vitro (Figure 2A), and subcutaneously implanted B16 *Hsd11b1^–/–^* cell tumors grew more slowly than control tumors (Figure 2, B and C). Whereas tumor de novo synthesis of glucocorticoids would be adrenal independent, glucocorticoid regeneration by 11β-HSD1 would depend on circulating DHC metabolized from adrenal corticosterone (25). Therefore, tumors were implanted in CYP11B1-deficient (*Cyp11b1^Actin–Cre^*) mice, which have low levels of circulating glucocorticoids (11) and thus reduced amounts of DHC for tumor cell 11β-HSD1 to recycle. B16 tumors grew more rapidly in WT than *Cyp11b1^Actin–Cre^* recipients (Supplemental Figure 2, B and C), consistent with a role for systemic steroids as substrates for intratumor glucocorticoid generation. Importantly, B16 control and *Hsd11b1^–/–^* tumors grew identically in *Cyp11b1^Actin–Cre^* mice (Supplemental Figure 2C), demonstrating that metabolites of adrenal-derived glucocorticoids are necessary for tumor-specific regeneration and promotion of tumor growth.

11β-HSD1 is generally thought to function in intracrine glucocorticoid signaling, meaning that the glucocorticoids generated by 11β-HSD1 bind and activate GR molecules within the same cell (25). Given that all nucleated cells express GR (encoded by *Nr3c1*), tumor cells would be the predicted targets of tumor cell–derived glucocorticoids. To determine whether the growth-promoting effect of *Hsd11b1* was due to direct alterations in tumor cell–intrinsic proliferation, we compared cell growth in vitro. *Hsd11b1* deletion had no effect on in vitro cell growth in the absence or presence of DHC or corticosterone (Figure 2D and Supplemental Figure 2D). To determine whether there was some adaptation in vivo, such as exposure to host-derived factors in the tumor microenvironment that rendered tumor cells susceptible to growth-promoting effects of glucocorticoids, CRISPR/Cas9 targeting was used to generate *Nr3c1*-deficient B16 cells (Supplemental Figure 2E). Both control and *Nr3c1*-deficient B16 cells exhibited identical growth in vivo (Figure 2E and Supplemental Figure 2F), demonstrating that tumor-derived glucocorticoids do not signal in an autocrine manner to promote growth.

The role of 11β-HSD1 in tumor growth was tested in other cell types. Deletion of *Hsd11b1* in Panc02 cells similarly prevented DHC conversion to corticosterone in vitro (Figure 2F) and reduced growth of subcutaneously implanted tumors in vivo (Figure 2, G and H). Although deletion of *Hsd11b1* in MC38 cells prevented in vitro corticosterone generation (Figure 2I), it had only a modest effect on in vivo tumor growth (Figure 2, J and K). Reasoning that this was due to moderate 11β-HSD1 activity, we generated MC38 cells that overexpressed *Hsd11b1*. These MC38 *Hsd11b1Tg* cells produced 4- to 5-fold more corticosterone in vitro than their empty vector–transduced counterparts (Figure 2L) and exhibited increased tumor growth in vivo (Figure 2, M and N).

This increased growth was not due to direct alterations in tumor cell–intrinsic proliferation, as *Hsd11b1* overexpression had no effect on in vitro cell growth in the absence or presence of DHC or corticosterone (Supplemental Figure 2G). Thus, the extent to which tumor cells can regenerate glucocorticoids from inactive precursors directly corresponds to their ability to grow in vivo.

### Tumor-infiltrating cells do not express functionally significant levels of Cyp11b1.

**Figure 3:**
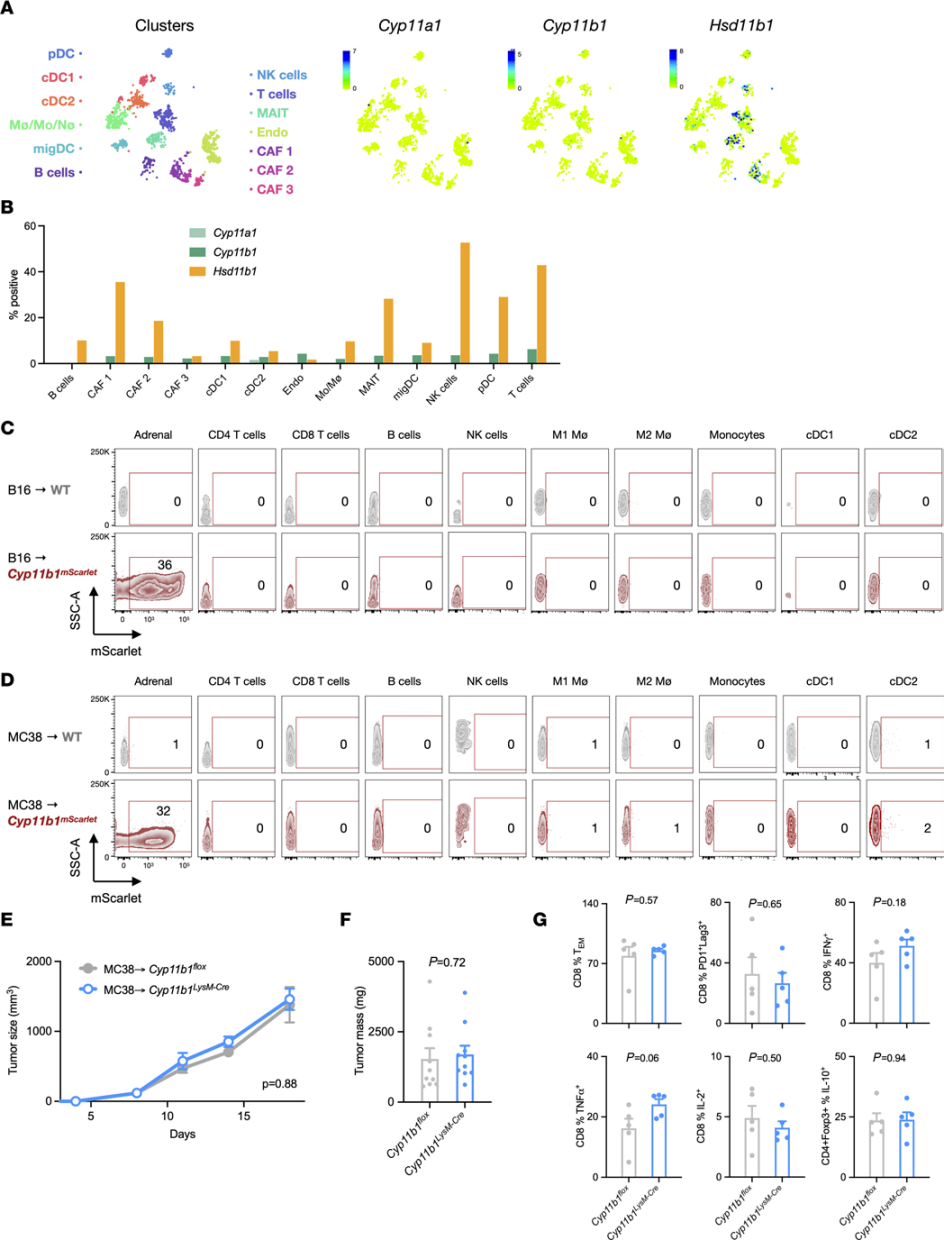
Lack of *Cyp11b1* expression and activity in tumor-infiltrating cells. (**A**) tSNE visualization of single-cell RNA profiles from mouse melanoma tumor-infiltrating cells. Data were obtained from the Wellcome Sanger mouse genomes project (https://www.sanger.ac.uk/data/mouse-genomes-project/) and visualized using the CELLxGENE platform. (**B**) Data from **A** presented as the percentages of cells within each cluster expressing any detectable transcripts of *Cyp11a1*, *Cyp11b1*, and *Hsd11b1*. (**C** and **D**) Flow cytometry analysis of adrenal cells and tumor-infiltrating cells from B16 cell–implanted WT and *Cyp11b1^mScarlet^* reporter mice (*n* = 3, 3) and MC38 cell–implanted WT and *Cyp11b1^mScarlet^* mice (*n* = 3,3). Representative contour plots (from left to right) show total live adrenal cells, and CD45^+^TCRβ^+^CD4^+^ T cells, CD45^+^TCRβ^+^CD8^+^ T cells, CD45^+^B220^+^ B cells, CD45^+^NK1.1^+^ NK cells, CD45^+^CD11b^+^F4/80^+^MHCII^+^ M1 macrophages, CD45^+^CD11b^+^F4/80^+^MHCII^–^ M2 macrophages, CD45^+^CD11b^+^F4/80^–^MHCII^+^ monocytes, CD45^+^CD11c^+^MHCII^+^CD8^+^ cDC1, and CD45^+^CD11c^+^MHCII^+^CD11b^+^ cDC2. Numbers show the percentages of mScarlet^+^ cells of each subset from WT (gray) and *Cyp11b1^mScarlet^* (red) mice. (**E**) MC38 tumor growth in control or *Cyp11b1^LysM–Cre^* (*Lyz2*-Cre) mice (*n* = 5, 5). Representative of 2 independent experiments. (**F**) MC38 tumor masses in control or *Cyp11b1^LysM–Cre^* mice (*n* = 10, 10). Data are pooled from 2 independent experiments. (**G**) Tumor-infiltrating T cell phenotypes in control or *Cyp11b1^LysM–Cre^* (*Lyz2*-Cre) mice (*n* = 5, 5). Representative of 2 independent experiments. Tumor growth was analyzed using rmANOVA with mouse genotype, sex, and experiment as factors. Tumor mass and cell frequencies were analyzed using ANOVA with mouse genotype and experiment as factors. Data are represented as means ± SEM with *P* values indicated in each panel. Supporting data are available in Supplemental Figure 3.

Although we found no evidence of *Cyp11b1* expression or activity in tumor cells, a recent study concluded that tumor-associated myeloid cells (TAMs) synthesize glucocorticoids de novo to promote tumor growth (20). The best evidence for the cellular source of glucocorticoids in tumors was provided by showing that mice with targeted deletion of *Cyp11a1*, which generates pregnenolone, a precursor of all steroids (Figure 1A), in myeloid cells (*Cyp11a1^LysM–Cre^* mice) had reduced tumor growth and increased CD8^+^ tumor-infiltrating lymphocytes (TILs) inflammatory cytokine production. Additionally, intratumor administration of metyrapone, which inhibits CYP11A1, CYP11B1, and 11β-HSD1 (26–29), reduced tumor growth. Reinterpretation of these data might instead suggest that myeloid CYP11A1–derived pregnenolone or vitamin D derivatives (30), rather than glucocorticoids, promoted tumor growth. However, it remained possible that TAM *Cyp11b1* could be a driver of tumor growth. We addressed this possibility in several ways. Single-cell transcriptomic data from mouse B16 melanomas (31) revealed that *Cyp11b1* was low to undetectable across tumor-infiltrating cell subsets (B cells, dendritic cells, endothelial cells, fibroblasts, macrophages, MAIT cells, neutrophils, NK cells, and T cells). In contrast, *Hsd11b1* was expressed by cancer-associated fibroblasts, T cells, MAIT cells, and a small proportion of macrophage/monocytes/neutrophils (Figure 3, A and B, and Supplemental Figure 3A). The detection of CYP11B1 protein is problematic because the available antibodies are nonspecific, perhaps due to crossreactivity with other P450 cytochrome enzymes (32). Therefore, we analyzed mice expressing an mScarlet-tagged CYP11B1 protein knocked in to the endogenous *Cyp11b1* locus (*Cyp11b1^mScarlet^* mice) to identify cells capable of de novo glucocorticoid synthesis (Taves, unpublished observations). Whereas CYP11B1^mScarlet^ was clearly detected in adrenal cells, we were unable to detect any CYP11B1^mScarlet^ in hematopoietic cells isolated from B16 (Figure 3C and Supplemental Figure 3B) or MC38 (Figure 3D and Supplemental Figure 3C) tumors implanted into *Cyp11b1^mScarlet^* mice. Nonetheless, it was conceivable that myeloid cells expressed low but sufficient CYP11B1 to produce biologically active amounts of glucocorticoids. To directly test the possible role of myeloid CYP11B1 in tumor growth, MC38 tumors were implanted into *Cyp11b1^LysM–Cre^* mice, in which *Cyp11b1* is deleted in myeloid cells and granulocytes. Unlike in the *Cyp11a1^LysM–Cre^* mice (20), tumors grew identically in control and *Cyp11b1^LysM–Cre^* mice (Figure 3, E and F) and no differences were observed in CD8^+^ TIL activation markers or inflammatory cytokine production (Figure 3G). Taken together, these results show that tumor-resident myeloid cells are unlikely to synthesize biologically relevant quantities of glucocorticoids from cholesterol.

### Tumor-generated glucocorticoids promote Treg and inhibit CD8^+^ T cell activity.

**Figure 4:**
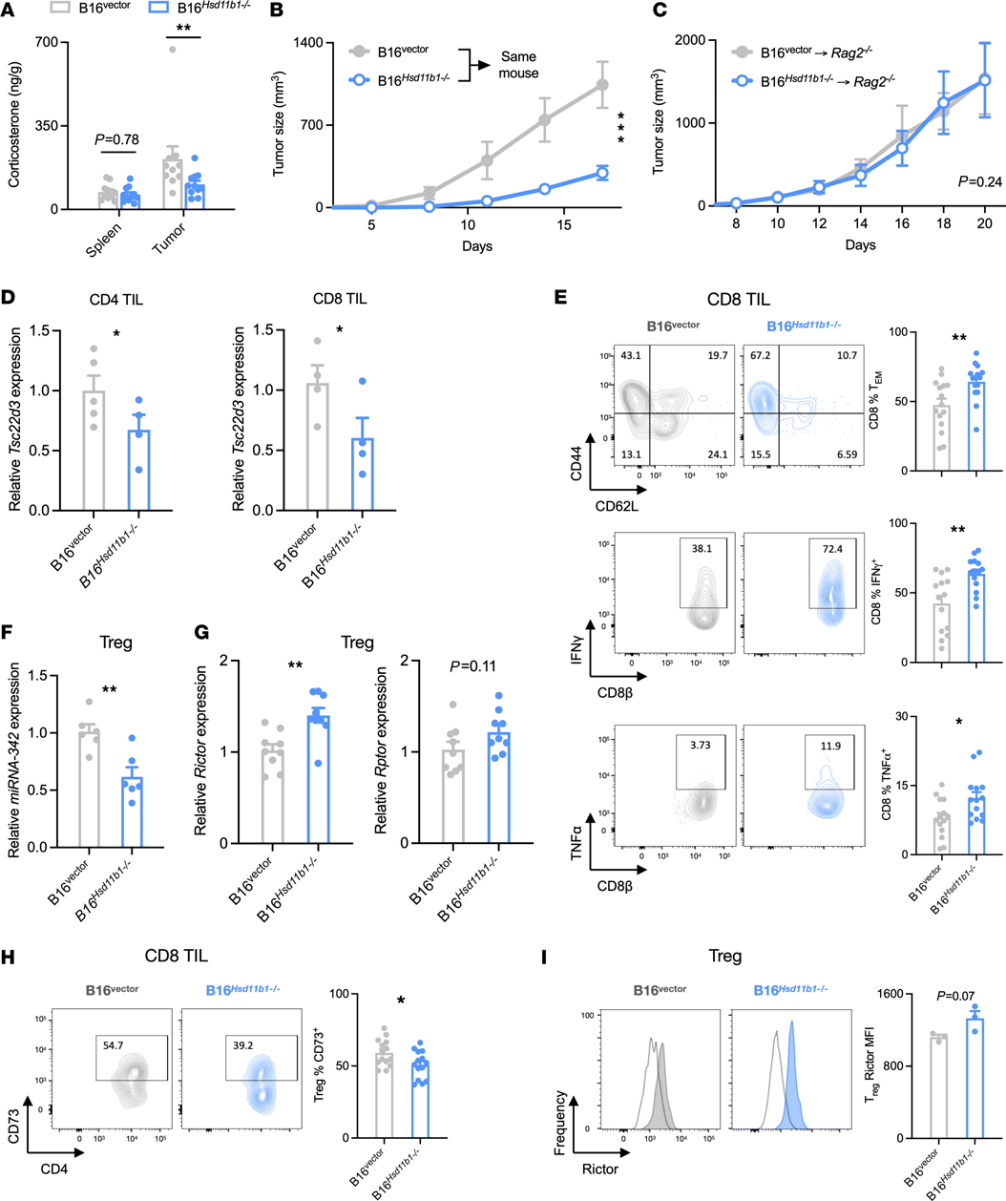
Tumor-generated glucocorticoids inhibit infiltrating CD8^+^ T cell activity and enhance Treg immunosuppression. (**A**) Corticosterone in spleen and tumor of B16 control or *Hsd11b1^–/–^* cell–implanted mice (*n* = 10, 11). Corticosterone was isolated with solid-phase extraction and quantified by immunoassay. (**B**) B16 control and *Hsd11b1^–/–^* tumor growth after bilateral implant into WT mice (*n* = 14). (**C**) B16 control and *Hsd11b1^–/–^* tumor growth in lymphopenic *Rag2^–/–^* mice (*n* = 6, 6). Representative of 2 experiments. (**D**) CD8^+^ and CD4^+^ T cell gene expression in B16 control and *Hsd11b1^–/–^* tumors (*n* = 5, 4). MACS-isolated cells were assayed via RT-qPCR normalized to *18S*. One B16^vector^ CD8 sample was contaminated and was discarded. (**E**) CD8^+^ T cell phenotypes in B16 control and *Hsd11b1^–/–^* tumors (*n* = 14, 14). Cells were isolated, restimulated, stained, and analyzed by flow cytometry. Data pooled from 3 experiments. (**F** and **G**) MicroRNA and mRNA expression in Tregs isolated from B16 control and *Hsd11b1^–/–^* tumors (*n* = 6, 6 and *n* = 9,9, respectively). FACS-isolated cells were assayed via RT-qPCR. Data pooled from 2 experiments. (**H**) Treg CD73 expression in B16 control and *Hsd11b1^–/–^* tumors (*n* = 14, 14). Cells were isolated, stained, and analyzed by flow cytometry. Data are pooled from 3 experiments. (**I**) Treg rictor in B16 control and *Hsd11b1^–/–^* tumors (*n* = 3, 3). Cells were isolated, stained, and analyzed by flow cytometry. Representative of 2 experiments. Tissue corticosterone was analyzed using rmANOVA with cell genotype, tissue, and mouse sex as factors. Tumor growth was analyzed using rmANOVA with cell genotype and mouse sex as factors. Gene and protein expression were analyzed using ANOVA with cell genotype, mouse sex, and experiment as factors, except for data in **I**, which were analyzed with an unpaired *t* test. Data are represented as means ± SEM. *P < 0.05; **P < 0.01; ***P < 0.001. Supporting data are available in Supplemental Figure 4.

The ability of tumor-generated glucocorticoids to affect tumor progression implies that there is a substantial amount of production in vivo. The contribution of tumor-generated glucocorticoids to the total tumor corticosterone content was determined by extracting tissue steroids from B16 tumors and quantifying corticosterone concentrations. WT or *Hsd11b1*-deficient B16 tumors were collected when they approached a diameter of 2 cm, as were spleens from the same animals. Corticosterone levels were similar in the spleens of control and *Hsd11b1^–/–^* B16 tumor-bearing animals (Figure 4A and Supplemental Figure 4B). In contrast, corticosterone concentrations were approximately 3-fold higher in tumors than in the spleen, an increase that was due to tumor-generated corticosterone because B16 *Hsd11b1^–/–^* tumors had levels similar to those in the spleen. Thus, tumor *Hsd11b1* expression is sufficient to substantially increase intratumor corticosterone levels. If tumor-derived corticosterone acts locally in a paracrine manner, rather than systemically, we would expect *Hsd11b1^–/–^* tumors to grow more slowly than controls even when implanted in the same animal. Indeed, B16 *Hsd11b1^–/–^* tumors grew more slowly than control tumors implanted on the contralateral flank of the same mouse (Figure 4B and Supplemental Figure 4C).

To determine whether lymphocytes are necessary for the growth-promoting action of tumor glucocorticoids, T and B cell–deficient *Rag2-*deficient mice were implanted with B16 control or matching *Hsd11b1^–/–^* tumors. Both tumors grew at the same rate (Figure 4C), demonstrating that lymphocytes are necessary targets (direct or indirect) of paracrine glucocorticoid signaling. Analysis of CD4^+^ and CD8^+^ TILs and other immune cell subsets found no difference in cell numbers between control and *Hsd11b1^–/–^* tumors, indicating that intratumor glucocorticoid signaling did not have a major effect on leukocyte trafficking into or expansion within the tumor (Supplemental Figure 4D), suggesting that their activity must be altered in the glucocorticoid-generating tumors. To determine whether T cells are directly signaled by tumor-derived glucocorticoids, mRNA was isolated from B16 control and *Hsd11b1^–/–^* tumor-infiltrating CD8^+^ and CD4^+^ T cells. Both had reduced expression of the glucocorticoid-responsive gene *Tsc22d3* (encoding GILZ) in the *Hsd11b1*-deficient tumors (Figure 4D). The absence of tumor cell 11β-HSD1 also resulted in CD8^+^ T cells with an increased fraction of effector memory cells (Tems) and increased production of the proinflammatory cytokines IFN-γ and TNF-α (Figure 4E). Treg function is regulated by mTORC signaling, with mTORC1/Raptor promoting and mTORC2/Rictor inhibiting Treg function (33–35). Recent findings have shown that glucocorticoids can suppress immune responses by Treg-specific induction of miRNA-342, which in turn downregulates *Rictor* and mTORC2 signaling (5). We indeed found that the Tregs infiltrating *Hsd11b1*-deficient tumors had decreased expression of miRNA-342 (Figure 4F) and increased expression of *Rictor* (but not *Rptor*) mRNA (Figure 4G). Tregs in *Hsd11b1*-deficient tumors also had reduced expression of the immunosuppressive CD73 surface protein and increased Rictor protein (Figure 4, H and I). These results raised the possibility that tumor-derived glucocorticoids drive the immunosuppressive capacity of tumor-infiltrating Tregs.

### Glucocorticoids suppress antitumor immunity by enhancing Treg function.

**Figure 5:**
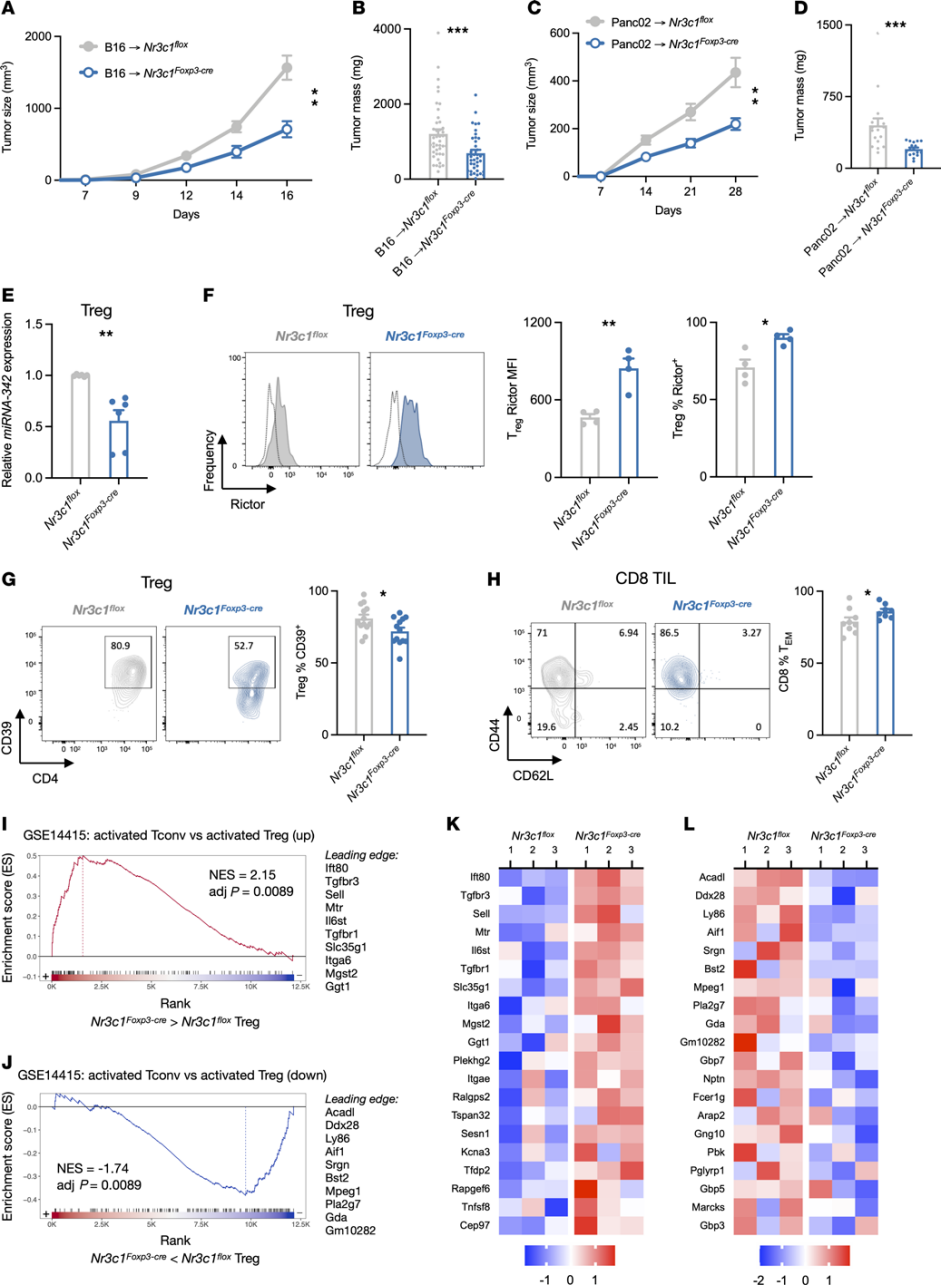
Glucocorticoids suppress antitumor immunity by enhancing Treg function. (**A**) B16 tumor growth in control or *Nr3c1^Foxp3-Cre^* mice (*n* = 10, 12). Representative of 6 experiments. (**B**) B16 tumor masses in control or *Nr3c1^Foxp3-Cre^* mice (*n* = 43, 37). Data pooled from 6 experiments. (**C**) Panc02 tumor growth in control or *Nr3c1^Foxp3-Cre^* mice (*n* = 6, 8). Representative of 2 experiments. (**D**) Panc02 tumor masses in control or *Nr3c1^Foxp3-Cre^* mice (*n* = 17, 17). Data pooled from 3 experiments. (**E**) Expression of miRNAs in FACS-sorted Tregs from B16 tumors implanted into control or *Nr3c1^Foxp3-Cre^* mice (*n* = 6, 6). Data pooled from 2 experiments. (**F**) Rictor expression in Tregs from B16 tumors of control or *Nr3c1^Foxp3-Cre^* mice (*n* = 4, 4). Tumor-infiltrating cells were isolated, stained, and analyzed by flow cytometry. Representative of 2 experiments. (**G**) Treg expression of CD39 in B16 tumors of control and *Nr3c1^Foxp3-Cre^* mice (*n* = 13, 13). Tumor-infiltrating cells were isolated, stained, and analyzed by flow cytometry. Data pooled from 3 experiments. (**H**) CD8^+^ T cell phenotypes in B16 tumors of control *Nr3c1^Foxp3-Cre^* mice (*n* = 8, 8). Tumor-infiltrating cells were isolated from tumors, stained, and analyzed by flow cytometry. Data pooled from 2 experiments. (**I** and **J**) Enrichment plots of genes upregulated (top) and downregulated (bottom) in GR-deficient versus control tumor-infiltrating Tregs compared with the gene set upregulated in activated Tconvs (CD4^+^Foxp3^–^ T cells) versus activated natural Tregs (CD4^+^Foxp3^+^ T cells). (**K** and **L**) Relative expression of the top 20 genes in GR-deficient and control tumor-infiltrating Tregs that paralleled increased expression in activated Tconvs versus activated Tregs (**K**) or decreased expression in activated Tconvs versus activated Tregs (**L**). Tumor growth was analyzed using rmANOVA with mouse genotype and sex as factors. Tumor masses, cell gene expression, and protein expression were analyzed using ANOVA with mouse genotype, sex, and experiment as factors, except for data in **F**, which was analyzed with an unpaired *t* test. Data are represented as means ± SEM. *P < 0.05; **P < 0.01; ***P < 0.001. Supporting data are available in Supplemental Figure 5.

The finding that tumor *Hsd11b1* expression upregulated markers of Treg activity prompted us to specifically investigate the role of Treg glucocorticoid signaling in tumor growth. B16 cells were implanted in mice in which the GR was specifically deleted in Tregs (*Nr3c1^Foxp3-Cre^* mice). We found that B16 tumors in *Nr3c1^Foxp3-Cre^* mice were slower-growing (Figure 5A) and smaller (Figure 5B) than those in WT mice. This was despite increased numbers of tumor-infiltrating Tregs in *Nr3c1^Foxp3-Cre^* mice, which was accompanied by increased numbers of CD8^+^ T cells and no change in the CD8^+^/Treg ratio (Supplemental Figure 5A). The same growth reduction was seen with Panc02 tumors implanted into *Nr3c1^Foxp3-Cre^* mice (Figure 5, C and D). To determine the degree to which tumor-generated glucocorticoids might affect antitumor responses in the absence of effects on Tregs, B16 control and *Hsd11b1*-deficient cells were implanted into *Nr3c1^Foxp3-Cre^* mice. Growth of *Hsd11b1*-deficient tumors was modestly reduced at late time points, but to a much lesser extent than in WT mice (Figure 2A), and did not reach statistical significance (*P* = 0.17) (Supplemental Figure 5B). These results indicate that tumor-derived glucocorticoids have a relatively small direct effect on the activity of non-Treg tumor-infiltrating cells and that Tregs are their primary target.

Because mice with T cell–specific or Treg-specific GR deletion have normal Treg numbers (6, 36), we hypothesized that reduced tumor growth in *Nr3c1^Foxp3-Cre^* mice was due to glucocorticoid-mediated enhancement of Treg function. Consistent with this, GR-deficient B16 tumor-infiltrating Tregs had reduced expression of miRNA-342 (Figure 5E), increased Rictor protein (Figure 5F), and reduced surface CD39 (Figure 5G). CD8^+^ T cells from these same tumors had a small but statistically significant increase in the fraction of CD8^+^CD44^hi^CD62L^lo^ Tem cells (Figure 5H), indicating that glucocorticoid signaling in Tregs acts upstream of CD8^+^ T cell activity. To better characterize the change in Treg state in response to glucocorticoids, tumor-infiltrating Tregs were isolated from B16 tumors implanted in control and *Nr3c1^Foxp3-Cre^* mice and RNA-Seq was performed. Expression of *Nr3c1* and the glucocorticoid-responsive genes *Tsc22d3*, *Dusp1*, and *Fkbp5* were reduced in *Nr3c1^Foxp3-Cre^* Tregs, which served as positive controls (Supplemental Figure 5C and Supplemental Table 1). We used gene set enrichment analysis (GSEA) against the immunological signatures database (C7) and found that 2 of the most significantly enriched gene sets (Supplemental Table 2) were found in activated T cells in a study comparing transcriptomes of ex vivo–activated conventional T cells (Tconv) (CD4^+^ Foxp3^–^ T cells) compared with ex vivo–activated natural Tregs (37). Specifically, tumor-infiltrating Treg genes that were overexpressed in the absence of GR matched genes that were upregulated in activated Tconv versus Treg (Figure 5, I and K). These included *Tgfbr1* (encoding TβRI)*,* a membrane-bound TGF-β receptor whose signaling reduces Treg suppression of Th1 cells by inhibiting T-bet expression (38), *Tgfbr3* (TβRIII), preferentially expressed in Tconv cells and downregulated during Treg differentiation (39), and *Il6st* (gp130), a marker of reduced suppressive capacity (40). Furthermore, tumor-infiltrating Treg genes that were underexpressed in the absence of GR also matched those that were downregulated in activated Tconvs versus Tregs (Figure 5, J and L). These included *Acadl* (LCAD), an enzyme involved in fatty acid oxidation supporting Treg proliferation and function (41), *Prdm1* (Blimp-1), an activator of *Il10* expression (42), *Prf1* (perforin), used by T and NK cells for target killing (43, 44), and *Srgn* (serglycin), a hematopoietic cell granule proteoglycan that forms the complex perforin-granzyme (45). A previous study identified differentially expressed genes in B16 tumor-infiltrating Tconvs versus Tregs (46). A comparison with tumor-infiltrating *Nr3c1^Foxp3-Cre^* Tregs again found enrichment for these Tconv genes (Supplemental Tables 3 and 4). Together, these analyses indicate that in the absence of glucocorticoid signaling, tumor Tregs acquired a transcriptional program more like that of activated Tconvs than activated Tregs. Tregs are therefore a key glucocorticoid target within the tumor microenvironment, and glucocorticoids help to maintain a regulatory phenotype.

### HSD11B1 is expressed in human tumors and correlates with signature T cell exhaustion and immunosuppressive genes.

**Figure 6:**
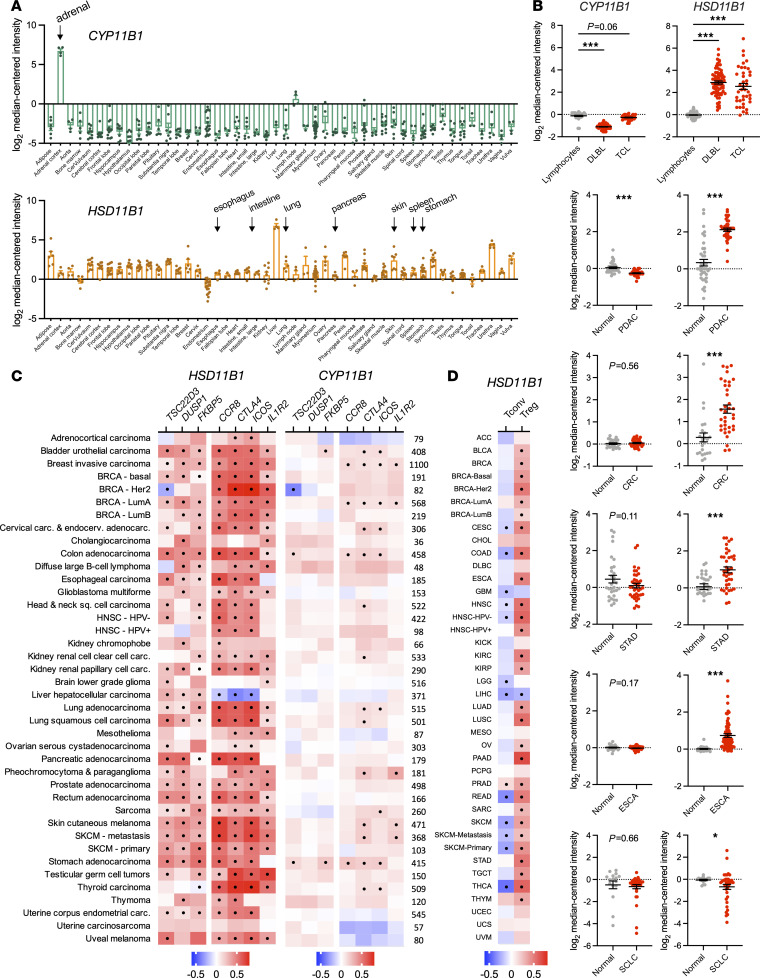
*HSD11B1* is expressed in human tumors and correlates with expression of T cell dysfunction and immunosuppression genes. (**A**) Relative gene expression of *CYP11B1* and *HSD11B1* in healthy adult human tissues (*n* = 3 to 27 per tissue). Data were acquired on Oncomine (https://www.oncomine.org/). (**B**) Relative gene expression of *CYP11B1* and *HSD11B1* in cancer versus normal tissues. Sample sizes were as follows: lymphocytes, 40; diffuse B cell lymphoma (DLBL), 70; T cell lymphoma (TCL), 40; normal pancreas, 39; pancreatic ductal adenocarcinoma (PDAC), 39; normal colon, 24; colorectal cancer (CRC), 36, normal stomach, 31; stomach adenocarcinoma (STAD), 38; normal esophagus, 28; esophageal adenocarcinoma (ESCA), 75; normal lung, 29; squamous cell lung carcinoma (SCLC), 87. Data acquired on Oncomine are represented as means ± SEM and were analyzed using ANOVA or unpaired *t* tests. Multiplicity-adjusted significance of *P < 0.05; ***P < 0.001. (**C**) Correlation between expression of *CYP11B1* (left) or *HSD11B1* (right) and glucocorticoid response genes (*TSC22D3, DUSP1, FKBP5*), and Treg marker genes (*CCR8, CTLA4, ICOS, IL1R2*) in human cancers. TCGA gene expression data were analyzed using TIMER2.0 and are presented as heatmaps showing the strength of the correlation (partial Spearman’s ρ) with significant correlations (*P* < 0.05 after adjusting for multiple comparisons) indicated with black circles. Sample sizes are shown at right. (**D**) Correlation between expression of *HSD11B1* with bulk RNA-Seq estimated frequency of tumor-infiltrating Tconvs and Tregs. TCGA gene expression data were analyzed using the quanTIseq algorithm on TIMER2.0 and are presented as heatmaps showing the strength of the correlation (partial Spearman’s ρ) with significant correlations (*P* < 0.05 after adjusting for multiple comparisons) indicated with black circles. Sample sizes are the same as in **D**. Supporting data are available in Supplemental Figure 6.

To determine whether 11β-HSD1-mediated glucocorticoid production is a potential target in clinical disease, its expression was examined across human tissues. Similarly to patterns seen in mouse tissues, *CYP11B1* expression was extremely low in all tissues other than the adrenals, whereas *HSD11B1* expression was widespread (Figure 6A). Importantly, *HSD11B1* but not *CYP11B1* expression was upregulated in cancer compared with matched healthy control tissues in a range of human tumors including lymphomas (B cell and T cell) and pancreatic, colorectal, stomach, and esophageal cancers (Figure 6B). Parallel to our findings in mouse tumor cell lines, *HSD11B1* was expressed at low levels in squamous cell lung cancer (Figure 6B). Across tumor types, *HSD11B1* expression correlated positively with expression of the glucocorticoid-responsive genes *TSD22D3* (GILZ), *DUSP1*, and *FKBP5* (Figure 6C). In contrast, glucocorticoid-responsive genes correlated poorly with *CYP11B1* (Figure 6C) or *CYP11A1* (Supplemental Figure 6B) expression, suggesting that any locally produced intratumor glucocorticoids would be primarily derived from regenerated metabolites rather than de novo synthesis. *HSD11B1* expression also positively correlated with Treg markers *CCR8, CTLA4, ICOS,* and *IL1R2* (Figure 6C) and T cell exhaustion markers *PDCD1*, *LAG3*, *HAVCR2*, and *TIGIT* (Supplemental Figure 6B). Furthermore, when we used gene signatures to estimate the frequency of tumor-infiltrating cells across tumor types (47), *HSD11B1* expression was negatively correlated with infiltrating Tconv frequency and positively correlated with infiltrating Treg frequency (Figure 6D). These patterns are consistent with the findings in mice and with a role for tumor *HSD11B1* in suppressing immunity and promoting growth of a variety of human cancers.

### Pharmacological inhibition of 11β-HSD1 reduces GEM tumor growth and enhances the response to anti–PD-1.

**Figure 7:**
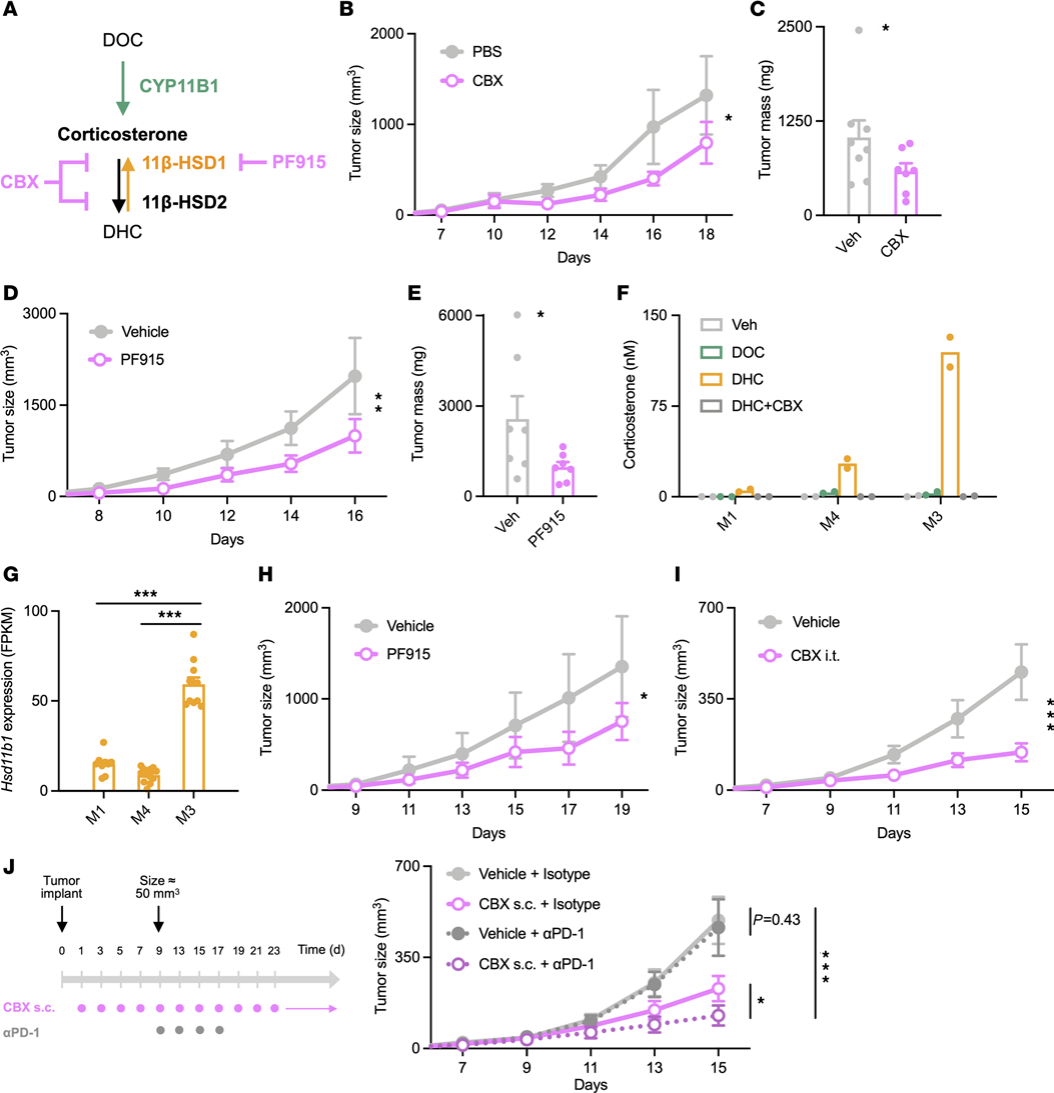
Pharmacological inhibition of 11β-HSD1 reduces tumor growth. (**A**) Inhibition of glucocorticoid generation by carbenoxolone and PF-915275. (**B**) B16 tumor growth in WT mice treated daily (beginning 24 hours after implant) with 50 mg/kg carbenoxolone or PBS (200 μL subcutaneous) (*n* = 3, 4). Representative of 2 experiments. (**C**) B16 tumor masses in PBS- or carbenoxolone-treated mice (*n* = 8, 7). Data pooled from 2 experiments. (**D**) B16 tumor growth in mice treated daily with 50 mg/kg PF-915275 or vehicle (200 μL subcutaneous) (*n* = 4, 4). Representative of 2 experiments. (**E**) B16 tumor masses in vehicle- or PF-915275–treated mice (*n* = 7, 7). Data pooled from 2 experiments. (**F**) Corticosterone production by 5 × 10^4^ M1, M4, or M3 mouse melanoma cells cultured 45 minutes with 100 μM carbenoxolone and then overnight with 100 nM DOC or DHC. Corticosterone in supernatants was quantified via immunoassay. Data show duplicate samples and are representative of 2 experiments. (**G**) RNA-Seq data from M1, M4, and M3 cells (*n* = 9, 12, 11 samples) (51) analyzed for relative *Hsd11b1* expression. (**H**) M3 tumor growth in mice treated daily with 50 mg/kg PF-915275 or vehicle (200 μL subcutaneous) (*n* = 4, 4). Representative of 2 experiments. (**I**) M3 tumor growth in WT mice treated with 6.25 mg/kg carbenoxolone or vehicle (50 μL intratumoral) (*n* = 7, 7) every 2 days (beginning day 9) for a total of 5 treatments. Representative of 2 experiments. (**J**) M3 tumor growth in mice treated daily with 6.25 mg/kg carbenoxolone or vehicle (200 μL subcutaneous) and treated with 10 mg/kg anti–PD-1 or rat IgG_2a_ isotype control (200 μL intraperitoneal). Vehicle/isotype, *n* = 10; vehicle/anti–PD-1, *n* = 10; carbenoxolone/isotype, *n* = 12; carbenoxolone anti–PD-1, *n* = 10 mice. Tumor growth was analyzed using rmANOVA with treatment and sex as factors. Tumor masses were analyzed using ANOVA with treatment, sex, and experiment as factors. Data are shown as mean ± SEM. *P < 0.05; **P < 0.01; ***P < 0.001.

Because genetic disruption of tumor-expressed *Hsd11b1* suppressed tumor growth in vivo, we asked whether administration of 11β-HSD1 inhibitors would have the same effect. B16 tumor-bearing mice were treated with carbenoxolone, an inhibitor of 11β-HSD1 and 11β-HSD2 (48–50), or PF-915275, a specific inhibitor of 11β-HSD1 (50, 51) (Figure 7A). Carbenoxolone (Figure 7, B and C) and PF-915275 (Figure 7, D and E) inhibited B16 tumor growth to a degree similar to that seen with B16 tumors lacking *Hsd11b1* expression. The role of 11β-HSD1 in melanoma was further explored using recently developed syngeneic cell lines from a genetically engineered melanoma model (GEM), M1, M4, and M3, which represent different stages of melanocyte differentiation and whose transcriptomes closely match those of major human melanoma subtypes. Specifically, M1 cells exhibit a neural crest–like phenotype and cluster with BRAF-mutant human melanomas, M4 cells have a transitory phenotype and cluster with RAS-mutant human melanomas, and M3 cells have a melanocytic phenotype and cluster with human triple-WT (BRAF/RAS/NF1-WT). These mouse cell lines also match the immunotherapy response profiles of their corresponding human tumors (52). None of the melanoma cell lines were able to convert DOC to corticosterone, indicating an absence of CYP11B1 activity, but all did convert DHC into corticosterone, demonstrating the presence of 11β-HSD1 (Figure 7F). M3 cells in particular converted all of the available DHC to corticosterone, with M4 and M1 cells producing progressively less, suggesting that 11β-HSD1 activity might increase as a function of melanocytic differentiation from neural crest-like (M1) to transitory (M4) to melanocytic (M3). Gene expression of *Hsd11b1* was correspondingly highest in M3 cells (Figure 7G). Notably, as with B16 cells, treatment with carbenoxolone (Figure 7H) or PF-915275 (Figure 7I) inhibited growth of M3 tumors in vivo. Together, these data demonstrate that 11β-HSD1 can be targeted with small-molecule reagents to inhibit the growth of *Hsd11b1*-expressing tumors.

The observation that 11β-HSD inhibition was sufficient to inhibit tumor growth raised the possibility that it might have additive or synergistic effects in combination with other antitumor treatments that target different molecular pathways. Because M3 melanoma cells are resistant to anti–PD-1 immune checkpoint blockade (52), we asked whether 11β-HSD inhibition could sensitize M3 tumors to anti–PD-1 therapy. Mice were implanted subcutaneously with M3 melanoma cells and treated with carbenoxolone, anti–PD-1, or both (Figure 7J). Anti–PD-1 alone had no effect on tumor growth, whereas systemic administration of carbenoxolone had a substantial effect (Figure 7J). Notably, anti–PD-1 also increased the effect of *Hsd11b1* deficiency on the growth of B16 tumors (Supplemental Figure 7). Therefore, in both tumor models, the combination of checkpoint blockade and 11β-HSD inhibition had a more substantial effect than either alone, suggesting that inhibiting local glucocorticoid regeneration may be a useful adjunct with classical immune checkpoint inhibitors.

## Discussion

In this study, we found that tumor cell–intrinsic expression of *Hsd11b1* allows generation of immunoregulatory glucocorticoids that act upon immune cells, especially Tregs, to suppress local antitumor immunity and promote tumor growth. Tumor cell *Hsd11b1* expression markedly increased intratumor corticosterone levels, reduced CD8^+^ TIL activity, and increased the immunosuppressive function of tumor-infiltrating Tregs. Of particular importance, pharmacological inhibition of 11β-HSD1 reduced tumor growth and sensitized tumors to immune checkpoint blockade. Together with data indicating *HSD11B1* expression and correlated exhaustion and immunosuppressive gene signatures across a range of human tumors, these results suggest that paracrine glucocorticoid signaling within the tumor microenvironment is an important mechanism by which some cancers evade antitumor immunity.

Natural and synthetic glucocorticoids are well known for their immunosuppressive actions (53) and are administered in clinical settings to control overzealous immune responses, such as when immunotherapies result in uncontrolled T cell activation. Glucocorticoids correspondingly promote growth of various tumors in mice and humans, and the discovery of extra-adrenal glucocorticoid production at sites of immune activation raised the possibility that tumor cells might produce them to suppress immune activation and function (54). This idea was supported by the finding of glucocorticoid production in vitro by tumor biopsies (23), but the in vivo relevance, as well as production pathways, cell sources, and cell targets of tumor-derived glucocorticoids have remained unclear. Our data show that a variety of murine tumor cell lines generate glucocorticoids in vitro (7 of 8 tested cell lines) and found that the same enzymatic pathway was used in all. Glucocorticoids can be synthesized de novo from cholesterol via a cascade of enzymes beginning with CYP11A1 and terminating with CYP11B1 or regenerated from circulating metabolites via 11β-HSD1 (Figure 1A). Recent studies have shown that CYP11A1, which converts cholesterol into the steroid pregnenolone, is expressed by T cells (21) and macrophages (20) and its expression in each of these cells functions to promote tumor growth. The tumor-promoting effect of CYP11A1 in macrophages and tumor-suppressing effect of intratumor metyrapone administration were proposed to be due to their respective actions in driving and inhibiting glucocorticoid synthesis (20). Our data, in contrast, indicate that CYP11B1 expression and activity in tumor cells and tumor-infiltrating cells is minimal and that 11β-HSD1 is the primary source of intratumor glucocorticoid production. Furthermore, the tumor-suppressing effect of metyrapone, provided as proof of de novo glucocorticoid synthesis, is in fact completely consistent with tumor cell glucocorticoid recycling, as metyrapone is a potent inhibitor of 11β-HSD1 (29, 55). T cell and macrophage–expressed CYP11A1 may instead promote tumor growth by generating pregnenolone that acts via a nonglucocorticoid signaling pathway, such as by activation of pregnane X receptors (PXRs) (21), or further pregnenolone conversion to progesterone, androgens, or estrogens, all of which can have immunomodulatory actions (56) and direct growth-promoting actions on tumor cells (57). CYP11A1 also produces vitamin D derivatives, secosteroids, which have a range of immunomodulatory activities (58). Our data further show that some tumor cells can generate high quantities of glucocorticoids, leading to a 2- to 3-fold increase in intratumor corticosterone concentrations. Because *Hsd11b1* expression is widespread in healthy mammalian tissues and upregulated in multiple human cancer types, its actions in driving tumor growth may be similarly widespread. The fact that *Hsd11b1* is also expressed by tumor-infiltrating immune cells might mean that intratumor glucocorticoid production also occurs at biologically relevant levels in tumors that themselves express little *Hsd11b1* and might contribute to a further glucocorticoid increase in those that do express higher *Hsd11b1*.

The GR is ubiquitously expressed, and tumor-derived glucocorticoids likely signal all or nearly all cells within the tumor microenvironment. Nonetheless, the results in this study show that T cells are the primary target of glucocorticoid immunosuppression, as tumor-derived glucocorticoids have little effect in lymphopenic mice. Glucocorticoids likely act at multiple levels to inhibit T cell antitumor immunity, including suppression of antigen-presenting cells, macrophages, and CD4^+^ helper and CD8^+^ effector T cells, and we indeed found that both CD4^+^ and CD8^+^ T cells are signaled by tumor-derived glucocorticoids. Our data suggest, however, that Tregs are a major effector of intratumor glucocorticoid-mediated immunosuppression because loss of Treg GR signaling contributed to a reduction in tumor growth similar to that seen with the loss of tumor *Hsd11b1*. The importance of Tregs as a target of tumor glucocorticoids is underscored by the minor effect of tumor *Hsd11b1* deficiency in the absence of Treg GR. This is consistent with studies demonstrating that glucocorticoid signaling is an important promoter of Treg function (5, 6) and that Tregs are a primary driver of glucocorticoid-mediated immunosuppression (5). Combined with the fact that Tregs can be essential for tumor growth (59, 60), glucocorticoid action in Tregs may be of central importance in tumor immune evasion.

Glucocorticoids, acting as systemic regulators of immune cell activity, are appreciated as both useful and problematic targets in cancer treatment. High doses of exogenous glucocorticoids can be lymphotoxic and are a mainstay in treatment of certain lymphomas (61). However, potent immunosuppression means that inhibition of glucocorticoid action is a preferred approach for promotion of immune responses in nonlymphoid solid tumors (20, 62–64). Such inhibition is problematic because systemic glucocorticoid insufficiency creates severe side effects and increased mortality (65). Intratumoral administration of aminoglutethimide (21), which inhibits synthesis of pregnenolone and estrogens (66), or metyrapone (20), which inhibits CYP11A1 and 11β-HSD1 production of corticosterone (29, 55), has been used to reduce tumor growth in vivo. These also inhibit adrenal glucocorticoid synthesis and reduce circulating glucocorticoids, and the potential for this and other actions outside of the tumor make them unsuitable for long-term use (67). Inhibitors of 11β-HSD1, in contrast, do not reduce systemic glucocorticoids levels or stress responses and are well tolerated, making them much safer for clinical use (68, 69). A number of 11β-HSD1–specific inhibitors are currently under investigation for treatment of disorders such as metabolic disease and diabetes (70–74). Our data suggest that 11β-HSD1 inhibition would have similar therapeutic value while maintaining systemic endocrine function relatively unperturbed. The option of systemic administration could also have the benefit of inhibiting 11β-HSD1 activity at metastatic sites. The fact that 11β-HSD1 inhibition sensitized tumors to checkpoint inhibition suggests that it could complement existing treatments, especially in 11β-HSD1–upregulated solid tumors in which this might play a major role in normalization of the tumor microenvironment, a goal for long-lasting tumor treatment (75). Inhibition of 11β-HSD1 might be particularly useful due to reduction in the activity of Tregs, which orchestrate a range of immunosuppressive characteristics within the tumor. Inhibition of 11β-HSD1 expression and glucocorticoid signaling in other immune cells would only be expected to further promote tumor immunity. Because *Hsd11b1* is widely expressed in both mouse and human tissues, 11β-HSD1 may be a therapeutic target in many cancers.

## Methods

### Cell culture.

B16.F10 cells were maintained in RPMI 1640 supplemented with 10 mM HEPES, 2 mM l-glutamine, 1 mM sodium pyruvate, 0.1 mM nonessential amino acids, 55 μM 2-mercaptoethanol, 50 μg/ml gentamicin, and 3% heat-inactivated FBS. EL-4, Panc02, and Lewis lung carcinoma cells were maintained in DMEM supplemented with 10 mM HEPES, 2 mM l-glutamine, 55 μM 2-mercaptoethanol, 50 μg/ml gentamicin, and 10% FBS. MC38 cells were maintained in DMEM supplemented with 10 mM HEPES, 2 mM l-glutamine, 1 mM sodium pyruvate, 0.1 mM nonessential amino acids, 55 μM 2-mercaptoethanol, 50 μg/ml gentamicin, and 10% FBS. M1, M3, and M4 melanoma cells were maintained in RPMI 1640 medium supplemented with 10 mM HEPES, 2 mM l-glutamine, 55 μM 2-mercaptoethanol, 50 μg/ml gentamicin, and 10% FBS. All cells were cultured at 37°C with 5% CO_2_.

### Cell-line generation.

Genetic deletion of *Hsd11b1* and *Nr3c1* (GR) were performed using CRISPR/Cas9 targeting with the pLentiCRISPRv2 plasmid as described (76). Briefly, complementary oligos encoding the sgRNA sequences were annealed, ligated into the pLentiCRISPRv2 plasmid, and transduced into Stbl3 cells. Ampicillin-resistant colonies were screened for integration by PCR, and plasmids (pLentiCRISPRv2, pVSVg, and psPAX2) were transfected into HEK293T cells. Approximately 1 million cells were transduced with lentiviral supernatants and expanded for 24 to 48 hours before 1 week of high-dose puromycin selection (B16, 2 μg/ml; EL-4, 4 μg/ml; Panc02, 4 μg/ml; MC38, 10 μg/ml) and maintenance under low-dose puromycin selection (B16, 1 μg/ml; EL-4, 8 μg/ml; Panc02, 2 μg/ml; MC38, 5 μg/ml). Three guides each were used to generate 3 polyclonal knockout cell populations (3 × *Hsd11b1^–/–^*, 3 × *Nr3c1^–/–^*), and these were screened in vitro for loss of enzyme activity (Hsd11b1) or stained to test for loss of protein expression (GR). The population with the greatest reduction in activity or protein was used for subsequent experiments. Control polyclonal cell populations were generated in the same way but without the sgRNA sequence cloned into the pLentiCRISPRv2 plasmid. Overexpression of *Hsd11b1* was performed by cloning the mouse *Hsd11b1* open-reading frame into the pMRX-IRES-Thy1.1 retroviral plasmid (77, 78), transducing HEK 293T cells, and infecting MC38 cells with retrovirus. Cells (control empty vector or *Hsd11b1*-encoding vector) with high levels of surface Thy1.1 reporter expression were stained and sorted. Cells were periodically checked for Thy1.1 expression (>95% Thy1.1^+^).

### Mice.

WT C57BL/6 mice and *Rag2^–/–^* mice were obtained from Jackson Laboratories. *Nr3c1^fl^* mice were generated by us (35) and crossed to mice expressing Foxp3 promoter–driven YFP/Cre recombinase expression for GR deletion specifically in Tregs. *Cyp11b1^fl^* mice were generated by us (11) and crossed to mice expressing Actb or LysM promoter-driven Cre recombinase (Jackson Laboratories) expression for global or macrophage-specific Cyp11b1 deletion, respectively. We generated and characterized (Taves, unpublished observations) mice expressing an mScarlet-tagged Cyp11b1 protein (*Cyp11b1^mScarlet^*). We did not detect any consistent sex differences in the effects of glucocorticoids on tumor growth and immune cell phenotype and therefore combined female and male mice for all analyses. Mice were kept on a 12-hour light/12-hour dark cycle, with ad libitum access to standard chow (NIH-31, Teklad).

### Tumor experiments.

Adherent cells were detached using 0.05% Trypsin-EDTA, washed with growth medium, resuspended in ice-cold PBS, passed through a 70 μm cell strainer, counted, and adjusted to 2.5 × 10^6^ cells/ml. Mice received subcutaneous injections of 200 μL (0.5 × 10^6^ cells) on the flank. Tumor length and width were measured using calipers and tumor volume calculated using the following formula: volume = length × width^2^. In some experiments, mice received daily subcutaneous injections of PF-915275 (25 mg/kg body weight in 200 μL PBS with 0.5% Tween 20), carbenoxolone (25 mg/kg body weight in 200 μL PBS), or vehicle, beginning the day after subcutaneous injection of tumor cells. Alternatively, carbenoxolone was also administered via intratumoral injection (50 μL). In other experiments, mice received intraperitoneal injections of 10 mg/kg anti–PD-1 (clone RMP1-14) or mouse IgG_2b_ isotype control (clone 2A3) in 100 μL PBS, beginning when the average tumor size in a cohort of mice reached approximately 50 mm^3^. When tumors approached 20 mm in length (or before), mice were euthanized with CO_2_, carcasses immediately chilled on wet ice, and tissues collected for further processing.

### Corticosterone production assay.

2.5 × 10^4^ Cells were aliquoted into a 96-well flat-bottom plate and incubated overnight in 150 μL of cell type–specific growth medium. The following day, cells (approximately 5 × 10^4^) were treated with carbenoxolone (100 μM) or metyrapone (100 μM), incubated for 30 to 45 minutes, and then treated with deoxycorticosterone (100 nM) or dehydrocorticosterone (100 nM) to a final volume of 200 μL. Cells were cultured for 24 hours, after which supernatants were collected, diluted in assay buffer, and corticosterone quantified by ELISA (Arbor Assays, K0015). Steroids were stored as 10 mM stocks in ethanol, kept in the dark at –20°C (deoxycorticosterone, corticosterone) or –80° (dehydrocorticosterone), and diluted immediately before use.

### Glucocorticoid bioactivity assay.

2.5 × 10^4^ Cells were aliquoted into a 96-well flat-bottom plate and incubated overnight in 150 μL of cell type–specific growth medium. The following day, cells (approximately 5 × 10^4^) were treated with 22R-hydroxycholesterol, deoxycorticosterone, or dehydrocorticosterone (100 nM each) in the presence of cAMP (8-bromo-cAMP, 1 mM), PMA (25 ng/mL), LPS (5 μg/mL), TNF-α (200 ng/mL), or IL-6 (200 ng/mL) in a final volume of 200 μL. Cells were cultured for 24 hours. Splenocytes from GR^GFP^ mice (79) were incubated in steroid-free medium for 2 hours, resuspended in B16- or MC38-conditioned media for 30 minutes, treated with permeabilization-fixation buffer (FACS buffer with 0.5% Triton X-100, 2% formaldehyde) for 25 minutes, washed with permeabilization buffer (FACS buffer with 0.1% Triton X-100), resuspended in FACS buffer (PBS containing 2% FBS and 0.05% sodium azide), and data acquired by flow cytometry. This assay selectively crosslinks and retains liganded (and therefore chromatin associated) GR molecules, and thus GR^GFP^ fluorescence provides a quantitative, ligand-agnostic test of glucocorticoid bioactivity (12).

### Tissue glucocorticoid quantification.

Tissues were dissected and immediately frozen in dry ice and stored at –80°C until processing. Frozen tumors were bisected and a piece from the interior of the tumor (approximately 50 mg) or piece of spleen (also approximately 50 mg) was removed and weighed. Tissues were briefly thawed on wet ice, diluted in 1 ml ice-cold 75% methanol, and immediately homogenized for 20 to 30 seconds with a Misonix Microson XL-2000 ultrasonic homogenizer. Homogenates were then incubated 30 minutes on wet ice, centrifuged at 10,000*g* for 10 minutes at 4°C, and placed in wet ice; 300 μL of supernatant was diluted with 5 ml water and immediately applied to C_18_ solid-phase extraction columns (Agilent Bond Elut C_18_ OH, 500 mg) that had been preconditioned with 3 ml hexane, 3 ml acetone, 3 ml methanol, and 5 ml water. After loading onto C_18_ columns, samples were washed with 5 ml 40% methanol, dried, and steroids eluted with 5 ml 90% methanol. Eluates were dried at 60°C in a Thermo SpeedVac vacuum centrifuge. Steroids were resuspended with 8 μL ethanol, briefly vortexed, diluted with 142 μL assay buffer, vortexed, and diluted 10-fold in assay buffer prior to corticosterone quantification by enzyme immunoassay.

### Cell isolation.

Tumors were dissected into ice-cold PBS, weighed, and minced in basic medium (RPMI 1640 containing 10 mM HEPES, 2 mM l-glutamine, 1 mM sodium pyruvate, and 0.1 mM nonessential amino acids) containing Liberase TL (Roche) and DNase I (MilliporeSigma). Tumors were digested with gentle agitation for 20 to 30 minutes at 37°C, then transferred into GentleMACS C tubes and dissociated for 60 seconds using the m_impTumor_01 program. Dissociated cells were run through a sieve, then a 100 μm cell strainer, and leukocytes enriched using Percoll gradient centrifugation (600 *g*) (44% and 67% Percoll layers). Cells were washed, counted, and then resuspended in FACS buffer for flow cytometry analysis or MACS buffer for T cell purification.

### Flow cytometry.

Approximately 5 × 10^6^ cells were aliquoted into tubes, washed with FACS buffer (PBS containing 2% FCS and 0.05% sodium azide), and resuspended in 100 μl of FACS buffer containing appropriate conjugated antibodies. Surface staining was done for 30 minutes on ice in the dark. Cells were washed, fixed, and permeabilized (using the eBioscience Fixation/Permeabilization Transcription Factor Staining Kit), and intracellular staining (where indicated) was performed overnight at 4°C. Cells were washed in permeabilization buffer and resuspended in FACS buffer, and data were acquired on a BD FACSsymphony or LSRFortessa. Data were analyzed using FlowJo (TreeStar).

### Treg sorting and RNA-Seq.

Tumor-infiltrating cells were stained with LIVE/DEAD Fixable Blue, PerCP-Cy5.5 anti-CD45.2, e450 anti-CD8β, and e780 anti-CD4, and cells to exclude (dump channel) were stained with PE-labeled antibodies against CD11b, CD11c, B220, NK1.1, and TCR-γδ. Tregs were identified as Dump^–^CD45.2^+^CD4^+^Foxp3-YFP^+^ and sorted with a BD FACSAria. Total RNA from sorted Tregs was extracted using RNeasy Mini Plus Kits (QIAGEN). Six mRNA-Seq samples were pooled and sequenced on a NextSeq 2000 P2 using SMARTer ultra-low input RNA v4-Nextera XT DNA and paired-end sequencing. Sample reads were trimmed for adapters and low-quality bases using Cutadapt, version 1.18 (80), before alignment to the reference genome (mm10) and transcript annotation using STAR, version 2.7.0f (81). Gene expression quantification analysis was performed using STAR, RSEM, version 1.3.1 (82), and the GENCODE M21 gene annotation. Downstream analysis and visualization were performed within the NIH Integrated Analysis Platform (NIDAP) using R programs developed on the Foundry platform (Palantir Technologies). Genes were filtered for low counts (<1 cpm), and quantile normalized prior to differential expression using limma-voom, version 3.38.3 (83). GSEA was performed using fGSEA, version 1.8.0 (84). A specific GSEA analysis against the RNA-Seq data set described previously (84) was performed as follows. RNA-Seq data from B16 tumor Tconv (Tu.Tc) and B16 tumor Treg (TITR) samples were obtained from GEO and DEG analysis performed to identify the 500 most upregulated and 500 most downregulated genes in Tconvs versus Tregs. These custom gene sets were added to the Hallmark gene set collection and evaluated was performed using L2P Pathway Overrepresentation Analysis.

### RT-qPCR.

Cultured tumor cells were detached and washed with ice-cold PBS. Tumor-infiltrating CD4^+^ and CD8^+^ T cells were enriched by positive selection using Miltenyi MicroBeads Kits with the indicated columns. Cells (T cells or cell lines) were counted and RNA extracted using RNeasy Plus columns (QIAGEN). RNA was quantified by NanoDrop and reverse transcribed using SuperScript IV (Invitrogen); gene expression was quantified in duplicate or triplicate using PowerUp SYBR Green (Applied Biosystems). For miRNA, RNA was isolated by using the Quick-RNA Microprep Kit (Zymo Research), cDNA prepared using the miRCURY LNA RT Kit (QIAGEN), and gene expression quantified in duplicate or triplicate using miRCURY LNA SYBR Green PCR Kits (QIAGEN). All were quantified on a QuantStudio 6 QPCR thermocycler and relative expression determined using the ΔΔCt method.

### Human tumor gene expression.

Gene expression data for human tumors was accessed using Oncomine, searching by gene using the “Cancer vs. Normal Analysis” tool. Correlations in multiple tumor types were analyzed using the TIMER to analyze data from The Cancer Genome Atlas (TCGA).

### Antibodies and reagents.

The following antibodies were used for flow cytometric analysis and cell sorting at a concentration of 1:100: eFluor 450–conjugated anti-CD44 (IM7, 48-0441-82), APC–eFluor 780–conjugated anti-CD62L (MEL-14, 47-0621-82), eFluor 450–conjugated anti-CD4 (GK1.5, 48-0041-82), APC–eFluor 780–conjugated anti-CD4 (RM4-5, 47-0042-82), PerCP–eFluor 710–conjugated anti-CD8β (H35-17.2, 46-0083-82), eFluor 450–conjugated anti-CD8β (H35-17.2, 48-0083-82), PE-conjugated anti–TCR-δγ (GL3, 12-5711-82), PE-conjugated anti-CD11b (M1/70, 12-0112-83), APC-conjugated anti-FOXP3 (FJK-16s, 17-5773-82), eFluor 450–conjugated anti-FOXP3 (FJK-16s, 48-5773-82), FITC-conjugated anti-FOXP3 (FJK-16s, 11-5773-82), FITC-conjugated anti–TNF-α (MP6-XT22, 11-7321-82), FITC-conjugated anti–IFN-γ (XMG1.2, 11-7311-82), and PE-conjugated anti–IFN-γ (XMG1.2, 11-7311-41) (from eBioscience); FITC-conjugated anti-CD4 (RM4-5, 553047), BV605-conjugated anti-CD4 (RM4-5, 563151), PerCP-Cy5.5–conjugated anti-CD45.2 (104, 552950), PE-conjugated anti-CD11c (HL3, 553802), PE-conjugated anti-CD45R/B220 (RA3-6B2, 553090), PE-conjugated anti-NK1.1 (PK136, 553165) (from BD Biosciences); Alexa Fluor 700–conjugated TCR-β chain (H57-597, 109224), PE/Cy7-conjugated anti-CD4 (RM4-5, 100527), Alexa Fluor 647–conjugated anti-CD39 (Duha59, 143808), Brilliant Violet 421–conjugated anti-CD73 (TY/11.8, 127217), Brilliant Violet 510–conjugated anti-CD45.2 (104, 109838) (from BioLegend); rabbit anti-mouse Rictor (53A2, 2114) (from Cell Signaling Technology); and goat anti-rabbit IgG (H+L) Alexa Fluor 546 (A11035) (from Invitrogen). The following reagents were used for flow cytometric analysis: Fixation/Permeabilization Solution Kit (BD Cytofix/Cytoperm, 554714) and Protein Transport Inhibitor containing Monensin (554724) (from BD Biosciences); Foxp3 /Transcription Factor Staining Buffer Set (00-5523-00) (from eBioscience); and LIVE/DEAD Fixable Blue Dead Cell Stain Kit (L23105) (from Invitrogen). PMA (P1585) and ionomycin (I0634) were purchased from MilliporeSigma and used for restimulation to induce cytokine production. For the enzyme activity assay, the following reagents were used: carbenoxolone disodium (C4790) and corticosterone (C2505) (from MilliporeSigma); DHC and DOC (from Steraloids); and Metyrapone (3292) (from Tocris). For quantitative reverse-transcriptase PCR (RT-qPCR), predesigned qPCR assays for *Rictor* (Mm.PT.58.7996582) and *Rptor* (Mm.PT.58.30504526) were purchased from IDT.

### Statistics.

Analyses were performed using GraphPad Prism (verision 9.0.0) and R (version 4.1.3). Data are presented as means with error bars indicating SEM, and significance was set at *P* < 0.05. Data were analyzed using within-subjects linear mixed-effects models where appropriate, using cell genotype, mouse genotype, experiment, and sex as factors as applicable. Experiments were performed multiple times as specified in figure legends.

### Study approval.

All protocols and procedures were approved by the NCI Animal Care and Use Committee and followed guidelines from the NIH *Guide for the Care and Use of Laboratory Animals* (National Academies Press, 2011).

### Data availability.

Cyp11b1^mScarlet^ mice will be provided to Jackson Laboratories. Sequence data were deposited in the NCBI’s Gene Expression Omnibus database (GEO GSE231303). Analysis pipelines are available from GitHub and can be accessed at https://github.com/NIDAP-Community/Tumors-produce-glucocorticoids-by-metabolite-recycling (branch name: main and commit ID: 52623cdd556943173d200d7d91c00d60a10f866). Values for all data points in graphs are reported in the Supporting Data Values file.

## Author contributions

MDT, SO, and JDA conceived and designed experiments. MDT, SO, MAT, and KMD performed experiments. TJM and MCC performed the bioinformatic analyses. MDT, SO, and JDA analyzed the data and wrote the paper. The authorship order of co–first authors was determined by chronological involvement in this study.

## Supplementary Material

Supplemental data

Supplemental tables 1-4

Supporting data values

## Version 1 07/20/2023

In-Press Preview

## Version 2 09/15/2023

Electronic publication

## References

[1] Sapolsky RM (2000). How do glucocorticoids influence stress responses? Integrating permissive, suppressive, stimulatory, and preparative actions. Endocr Rev.

[2] Jamieson AM (2010). Influenza virus-induced glucocorticoids compromise innate host defense against a secondary bacterial infection. Cell Host Microbe.

[3] Kugler DG (2013). CD4+ T cells are trigger and target of the glucocorticoid response that prevents lethal immunopathology in toxoplasma infection. J Exp Med.

[4] Taves MD, Ashwell JD (2021). Glucocorticoids in T cell development, differentiation and function. Nat Rev Immunol.

[5] Kim D (2020). Anti-inflammatory roles of glucocorticoids are mediated by Foxp3^+^ regulatory T cells via a miR-342-dependent mechanism. Immunity.

[6] Rocamora-Reverte L (2019). Glucocorticoid receptor-deficient Foxp3^+^ Regulatory T cells fail to control experimental inflammatory bowel disease. Front Immunol.

[7] Hamden JE (2019). Measurement of 11-dehydrocorticosterone in mice, rats and songbirds: Effects of age, sex and stress. Gen Comp Endocrinol.

[8] Salehzadeh M (2022). Glucocorticoid production in lymphoid organs: acute effects of lipopolysaccharide in neonatal and adult mice. Endocrinology.

[9] Morton NM (2004). Novel adipose tissue–mediated resistance to diet-induced visceral obesity in 11β-hydroxysteroid dehydrogenase type 1–deficient mice. Diabetes.

[10] Taves MD (2015). Steroid profiling reveals widespread local regulation of glucocorticoid levels during mouse development. Endocrinology.

[11] Mittelstadt PR (2018). Cutting edge: de novo glucocorticoid synthesis by thymic epithelial cells regulates antigen-specific thymocyte selection. J Immunol.

[12] Taves MD (2019). Single-cell resolution and quantitation of targeted glucocorticoid delivery in the thymus. Cell Rep.

[13] Vacchio MS (1994). Steroid production in the thymus: implications for thymocyte selection. J Exp Med.

[14] Cima I (2004). Intestinal epithelial cells synthesize glucocorticoids and regulate T cell activation. J Exp Med.

[15] Noti M (2010). TNF suppresses acute intestinal inflammation by inducing local glucocorticoid synthesis. J Exp Med.

[16] Phan TS (2021). Keratinocytes control skin immune homeostasis through de novo-synthesized glucocorticoids. Sci Adv.

[17] Slominski A (2005). CRH stimulation of corticosteroids production in melanocytes is mediated by ACTH. Am J Physiol Endocrinol Metab.

[18] Vukelic S (2011). Cortisol synthesis in epidermis is induced by IL-1 and tissue injury. J Biol Chem.

[19] García-Nieto PE (2019). The somatic mutation landscape of the human body. Genome Biol.

[20] Acharya N (2020). Endogenous glucocorticoid signaling regulates CD8^+^ T cell differentiation and development of dysfunction in the tumor microenvironment. Immunity.

[21] Mahata B (2020). Tumors induce de novo steroid biosynthesis in T cells to evade immunity. Nat Commun.

[22] Cirillo N (2017). Characterisation of the cancer-associated glucocorticoid system: key role of 11β-hydroxysteroid dehydrogenase type 2. Br J Cancer.

[23] Sidler D (2011). Colon cancer cells produce immunoregulatory glucocorticoids. Oncogene.

[24] Su AI (2002). Large-scale analysis of the human and mouse transcriptomes. Proc Natl Acad Sci U S A.

[25] Chapman K (2013). 11β-hydroxysteroid dehydrogenases: intracellular gate-keepers of tissue glucocorticoid action. Physiol Rev.

[26] Ahmad S (2017). Molecular docking reveals the potential of phthalate esters to inhibit the enzymes of the glucocorticoid biosynthesis pathway. J Appl Toxicol.

[27] Hostettler N (2012). Local glucocorticoid production in the mouse lung is induced by immune cell stimulation. Allergy.

[28] Johansson MK (2002). Effects of 3-MeSO2-DDE and some CYP inhibitors on glucocorticoid steroidogenesis in the H295R human adrenocortical carcinoma cell line. Toxicol In Vitro.

[29] Sampath-Kumar R (1997). Metyrapone is a competitive inhibitor of 11beta-hydroxysteroid dehydrogenase type 1 reductase. J Steroid Biochem Mol Biol.

[30] Slominski AT (2021). Editorial: steroids and secosteroids in the modulation of inflammation and immunity. Front Immunol.

[31] Davidson S (2020). Single-cell RNA sequencing reveals a dynamic stromal niche that supports tumor growth. Cell Rep.

[32] Taves MD et al (2023). Aire drives steroid hormone biosynthesis by medullary thymic epithelial cells. Sci Immunol.

[33] Charbonnier LM (2019). Functional reprogramming of regulatory T cells in the absence of Foxp3. Nat Immunol.

[34] Delgoffe GM (2011). The kinase mTOR regulates the differentiation of helper T cells through the selective activation of signaling by mTORC1 and mTORC2. Nat Immunol.

[35] Zeng H (2013). mTORC1 couples immune signals and metabolic programming to establish T(reg)-cell function. Nature.

[36] Mittelstadt PR (2012). Thymocyte responsiveness to endogenous glucocorticoids is required for immunological fitness. J Clin Invest.

[37] Haribhai D (2009). A central role for induced regulatory T cells in tolerance induction in experimental colitis. J Immunol.

[38] Konkel JE (2017). Transforming growth factor-β signaling in regulatory T cells controls T helper-17 cells and tissue-specific immune responses. Immunity.

[39] Ortega-Francisco S (2017). TβRIII is induced by TCR signaling and downregulated in FoxP3^+^ regulatory T cells. Biochem Biophys Res Commun.

[40] Bin Dhuban K (2019). Signaling through gp130 compromises suppressive function in human FOXP3+ regulatory T cells. Front Immunol.

[41] Kempkes RWM (2019). Metabolic pathways involved in regulatory T cell functionality. Front Immunol.

[42] Cretney E (2011). The transcription factors Blimp-1 and IRF4 jointly control the differentiation and function of effector regulatory T cells. Nat Immunol.

[43] Cao X (2007). Granzyme B and perforin are important for regulatory T cell-mediated suppression of tumor clearance. Immunity.

[44] Grossman WJ (2004). Human T regulatory cells can use the perforin pathway to cause autologous target cell death. Immunity.

[45] Metkar SS (2002). Cytotoxic cell granule-mediated apoptosis: perforin delivers granzyme B-serglycin complexes into target cells without plasma membrane pore formation. Immunity.

[46] Magnuson AM (2018). Identification and validation of a tumor-infiltrating Treg transcriptional signature conserved across species and tumor types. Proc Natl Acad Sci U S A.

[47] Finotello F (2019). Molecular and pharmacological modulators of the tumor immune contexture revealed by deconvolution of RNA-seq data. Genome Med.

[48] Brown RW (1996). Cloning and production of antisera to human placental 11β-hydroxysteroid dehydrogenase type 2. Biochem J.

[49] Hult M (1998). Selective inhibition of human type 1 11beta-hydroxysteroid dehydrogenase by synthetic steroids and xenobiotics. FEBS Lett.

[50] Zhang X (2009). 4-(Phenylsulfonamidomethyl)benzamides as potent and selective inhibitors of the 11beta-hydroxysteroid dehydrogenase type 1 with efficacy in diabetic ob/ob mice. Bioorg Med Chem Lett.

[51] Bhat BG (2008). Demonstration of proof of mechanism and pharmacokinetics and pharmacodynamic relationship with 4′-cyano-biphenyl-4-sulfonic acid (6-amino-pyridin-2-yl)-amide (PF-915275), an inhibitor of 11β-hydroxysteroid dehydrogenase type 1, in cynomolgus monkeys. J Pharmacol Exp Ther.

[52] Pérez-Guijarro E (2020). Multimodel preclinical platform predicts clinical response of melanoma to immunotherapy. Nat Med.

[53] Cain DW, Cidlowski JA (2017). Immune regulation by glucocorticoids. Nat Rev Immunol.

[54] Taves MD (2011). Extra-adrenal glucocorticoids and mineralocorticoids: evidence for local synthesis, regulation, and function. Am J Physiol Endocrinol Metab.

[55] Taves MD (2016). Lymphoid organs of neonatal and adult mice preferentially produce active glucocorticoids from metabolites, not precursors. Brain Behav Immun.

[56] Brown MA, Su MA (2019). An inconvenient variable: sex hormones and their impact on T cell responses. J Immunol.

[57] Boibessot C, Toren P (2018). Sex steroids in the tumor microenvironment and prostate cancer progression. Endocr Relat Cancer.

[58] Postlethwaite AE (2021). 20*S*-hydroxyvitamin D3, a secosteroid produced in humans, is anti-inflammatory and inhibits murine autoimmune arthritis. Front Immunol.

[59] Klages K (2010). Selective depletion of Foxp3+ regulatory T cells improves effective therapeutic vaccination against established melanoma. Cancer Res.

[60] Kline J (2008). Homeostatic proliferation plus regulatory T-cell depletion promotes potent rejection of B16 melanoma. Clin Cancer Res.

[61] Lin KT, Wang LH (2016). New dimension of glucocorticoids in cancer treatment. Steroids.

[62] Maeda N (2019). Glucocorticoids potentiate the inhibitory capacity of programmed cell death 1 by up-regulating its expression on T cells. J Biol Chem.

[63] Xing K (2015). Dexamethasone enhances programmed cell death 1 (PD-1) expression during T cell activation: an insight into the optimum application of glucocorticoids in anti-cancer therapy. BMC Immunol.

[64] Yang H (2019). Stress-glucocorticoid-TSC22D3 axis compromises therapy-induced antitumor immunity. Nat Med.

[65] Ngaosuwan K (2021). Increased mortality risk in patients with primary and secondary adrenal insufficiency. J Clin Endocrinol Metab.

[66] Santen RJ, Misbin RI (1981). Aminoglutethimide: review of pharmacology and clinical use. Pharmacotherapy.

[67] Hughes KA (2008). 11-Beta-hydroxysteroid dehydrogenase type 1 (11beta-HSD1) inhibitors in type 2 diabetes mellitus and obesity. Expert Opin Investig Drugs.

[68] Scott JS (2014). Medicinal chemistry of inhibitors of 11β-hydroxysteroid dehydrogenase type 1 (11β-HSD1). J Med Chem.

[69] Wamil M, Seckl JR (2007). Inhibition of 11beta-hydroxysteroid dehydrogenase type 1 as a promising therapeutic target. Drug Discov Today.

[70] Ajjan RA (2022). Oral 11β-HSD1 inhibitor AZD4017 improves wound healing and skin integrity in adults with type 2 diabetes mellitus: a pilot randomized controlled trial. Eur J Endocrinol.

[71] Bianzano S (2021). Safety, tolerability, pharmacokinetics and pharmacodynamics of single oral doses of BI 187004, an inhibitor of 11beta-hydroxysteroid dehydrogenase-1, in healthy male volunteers with overweight or obesity. Clin Diabetes Endocrinol.

[72] Hardy RS (2021). 11βHSD1 inhibition with AZD4017 improves lipid profiles and lean muscle mass in idiopathic intracranial hypertension. J Clin Endocrinol Metab.

[73] Oda S (2021). An Open-label phase I/IIa Clinical Trial of 11β-HSD1 inhibitor for cushing’s syndrome and autonomous cortisol secretion. J Clin Endocrinol Metab.

[74] Othonos N (2023). 11β-HSD1 inhibition in men mitigates prednisolone-induced adverse effects in a proof-of-concept randomised double-blind placebo-controlled trial. Nat Commun.

[75] Aktipis CA (2013). Life history trade-offs in cancer evolution. Nat Rev Cancer.

[76] Sanjana NE (2014). Improved vectors and genome-wide libraries for CRISPR screening. Nat Methods.

[77] Magen A (2019). Single-cell profiling defines transcriptomic signatures specific to tumor-reactive versus virus-responsive CD4^+^ T cells. Cell Rep.

[78] Saitoh T (2002). Lymphotoxin-beta receptor mediates NEMO-independent NF-kappaB activation. FEBS Lett.

[79] Brewer JA (2002). Green fluorescent protein-glucocorticoid receptor knockin mice reveal dynamic receptor modulation during thymocyte development. J Immunol.

[80] Martin M (2011). Cutadapt removes adapter sequences from high-throughput sequencing reads. EMBnet J.

[81] Dobin A (2013). STAR: ultrafast universal RNA-seq aligner. Bioinformatics.

[82] Li B (2011). RSEM: accurate transcript quantification from RNA-Seq data with or without a reference genome. BMC Bioinformatics.

[83] Ritchie ME (2015). limma powers differential expression analyses for RNA-sequencing and microarray studies. Nucleic Acids Res.

