# Supplementary material for: Tumors produce glucocorticoids by metabolite recycling, not synthesis, and activate Tregs to promote growth
Source: J Clin Invest. 2023 Sep 15;133(18):e164599. doi: 10.1172/JCI164599 (PMC10503810; doi:10.1172/JCI164599)
Supplement: Supplemental data [file jci-133-164599-s024.pdf]

FIGURE S1

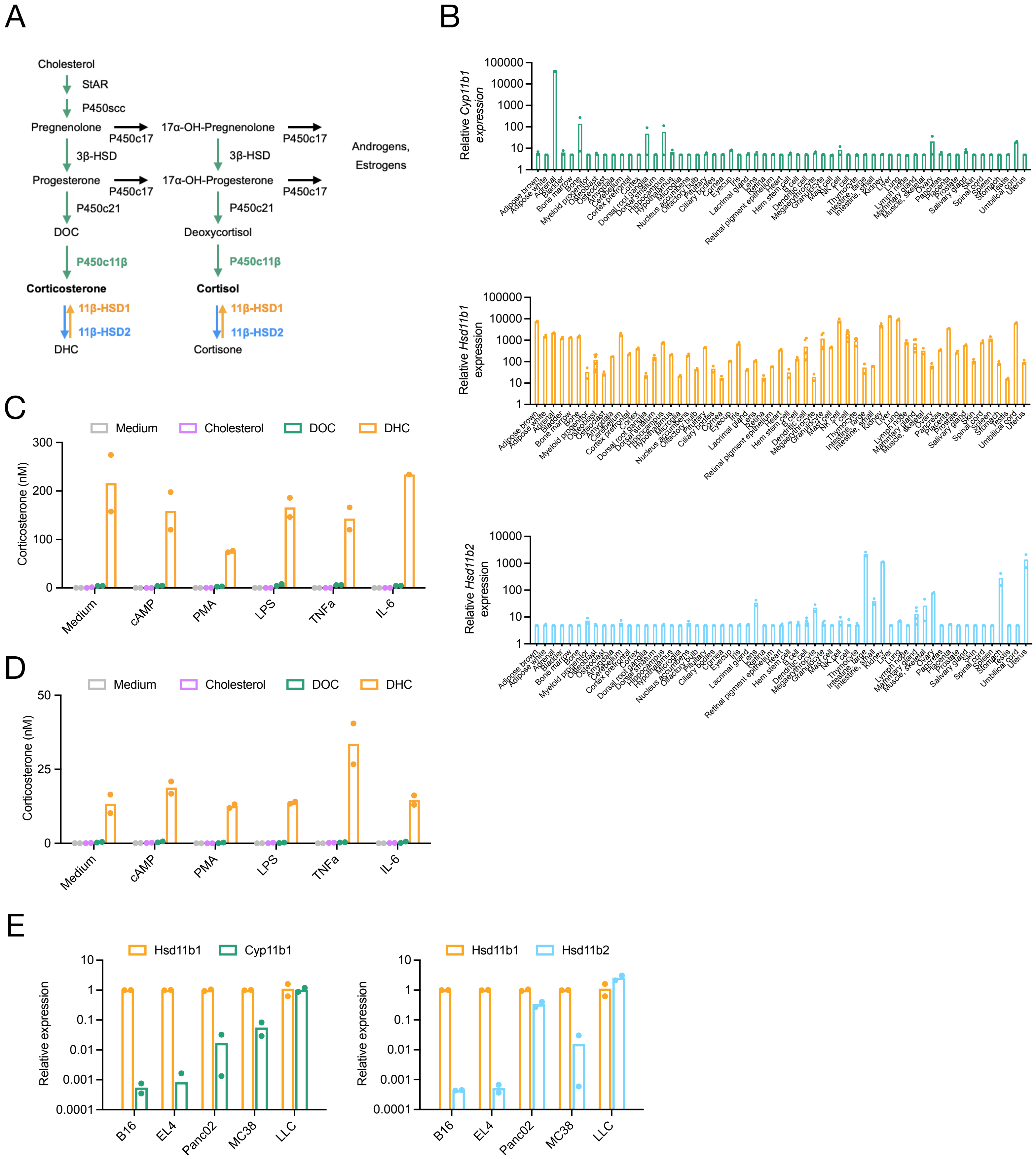

FIGURE S1 (related to Figure 1). Widespread glucocorticoid generation via 11β-HSD1 activity in healthy tissues and tumor cells.
(**A**) Simplified glucocorticoid synthetic pathway. Green arrows indicate enzyme-medicated steroid conversions required for *de novo* corticosterone biosynthesis from cholesterol, including corticosterone generation from its immediate precursor 11-deoxycorticosterone (DOC) by P450c11β (*Cyp11b1*), orange indicates corticosterone generation from inactive metabolite 11-dehydrocorticosterone (DHC) by 11β-HSD1 (*Hsd11b1*), and blue indicates corticosterone inactivation to DHC by 11β0-HSD1 (*Hsd11b2*).
(**B**) Relative gene expression of *Cyp11b1*, *Hsd11b1*, and *Hsd11b2* in adult mouse tissues. Data were acquired from BioGPS and asterisks indicate expression higher than that of the lowest tissue.
(**C,D**) Bioactive glucocorticoid production by (**C**) B16 cells and (**D**) MC38 cells. 5×10^4 tumor cells were cultured for cultured for 24 h with 100 nM 22R-hydroxycholesterol (cholesterol), deoxycorticosterone (DOC), or dehydrocorticosterone (DHC) to test for CYP11A1, CYP11B1, or 11β-HSD1 activity. Cells were also treated with cAMP, PMA, LPS, TNFα, or IL-6 to test for induction of glucocorticoid production. GR^{GFP} mouse splenocytes were resuspended in B16 or MC38-conditioned medium from various treatments and glucocorticoid activity quantified using a ligand titration assay (Taves et al., 2019).
(**E**) Relative gene expression of *Cyp11b1*, *Hsd11b1*, and *Hsd11b2* in mouse tumor cell lines. Total RNA was extracted from tumor cell lines and relative expression quantified via RT-qPCR, normalized to *18S* RNA expression.
Data are presented as means of duplicate samples (**B**) or wells (**C-E**).

FIGURE S2

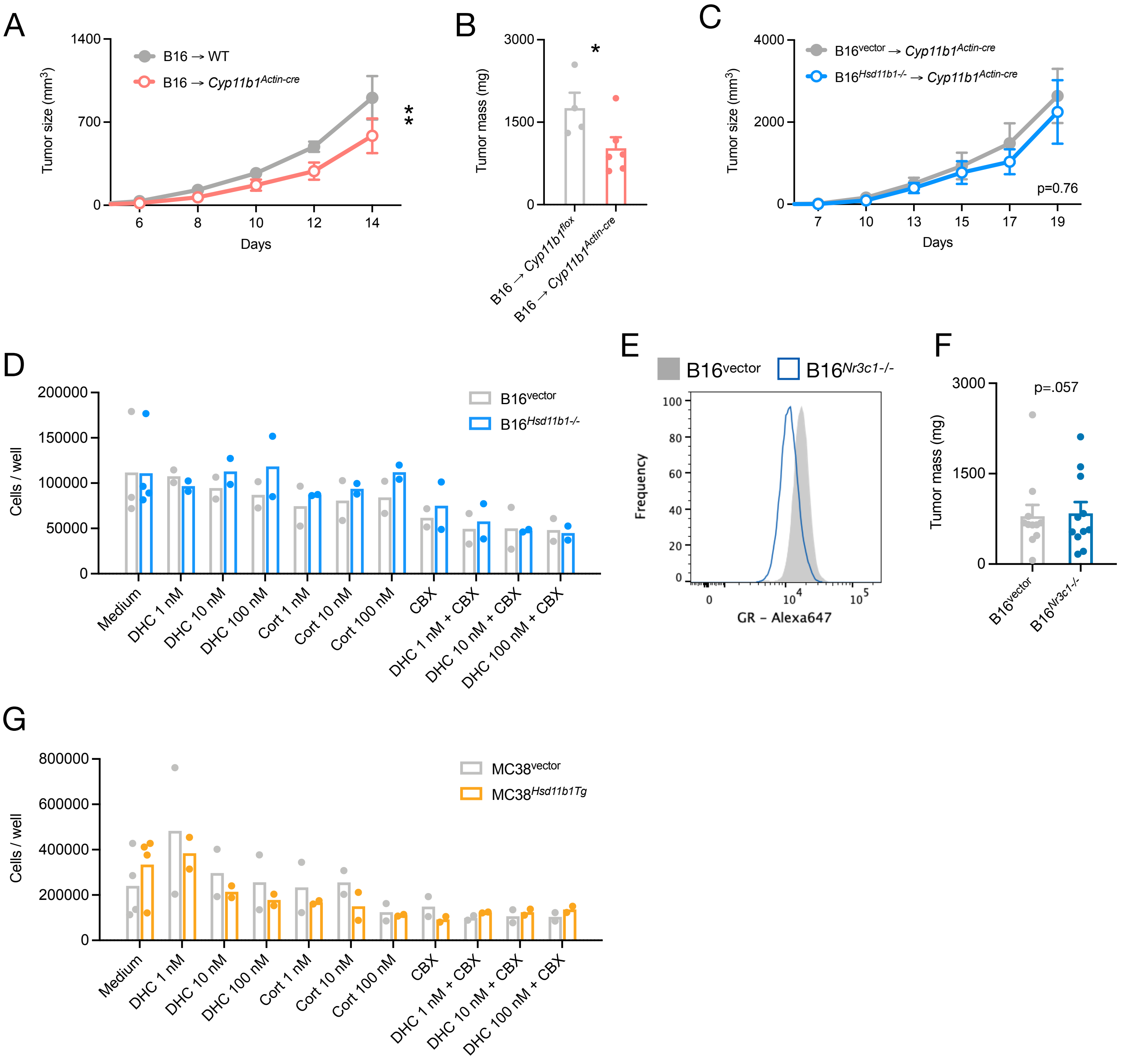

FIGURE S2 (related to Figure 2). 11β-HSD1 expression by cancer cells promotes tumor growth *in vivo*.

(**A**) Tumor growth of B16 cells implanted into control or *Cyp11b1*$^{Actin\text{-}cre}$ mice. Mice were implanted with 0.5×10^6 B16 control or *Hsd11b1-/-* cells, until tumors reach a diameter of 20 mm. Representative data are shown from 1 of 2 experiments.
(**B**) B16 tumor masses in control and *Cyp11b1*$^{Actin\text{-}cre}$ recipient mice. Representative of 2 experiments.
(**C**) Tumor growth of B16 control or *Hsd11b1-/-* cells implanted into *Cyp11b1*$^{Actin\text{-}cre}$ mice. Representative ata are shown from one of at least two experiments.
(**D, G**) B16 (D) control and *Hsd11b1-/-* cells or MC38 (G) control and *Hsd11b1Tg* cell numbers after 72 h in standard growth medium or medium supplemented with 100 nM DHC or corticosterone.
(**E**) Flow cytometry staining of the gucocorticoid receptor (GR) in B16^{vector} and B16$^{Nr3c1\text{-/-}}$ cells. Cultured cells were detached, fixed with BD cytofix to preserve total intracellular GR protein, stained with Alexa 647-conjugated anti-GR antibody, and data acquired by flow cytometry.
(**F**) Tumor masses of B16^{vector} and B16$^{Nr3c1\text{-/-}}$ tumors implanted into control mice. Data are pooled from two experiments.
Data are presented as means of duplicate samples (D, G) or mean ± SEM (B, C, F) with significance of p<0.05, <0.01, <0.001 indicated as *, **, ***.

FIGURE S3

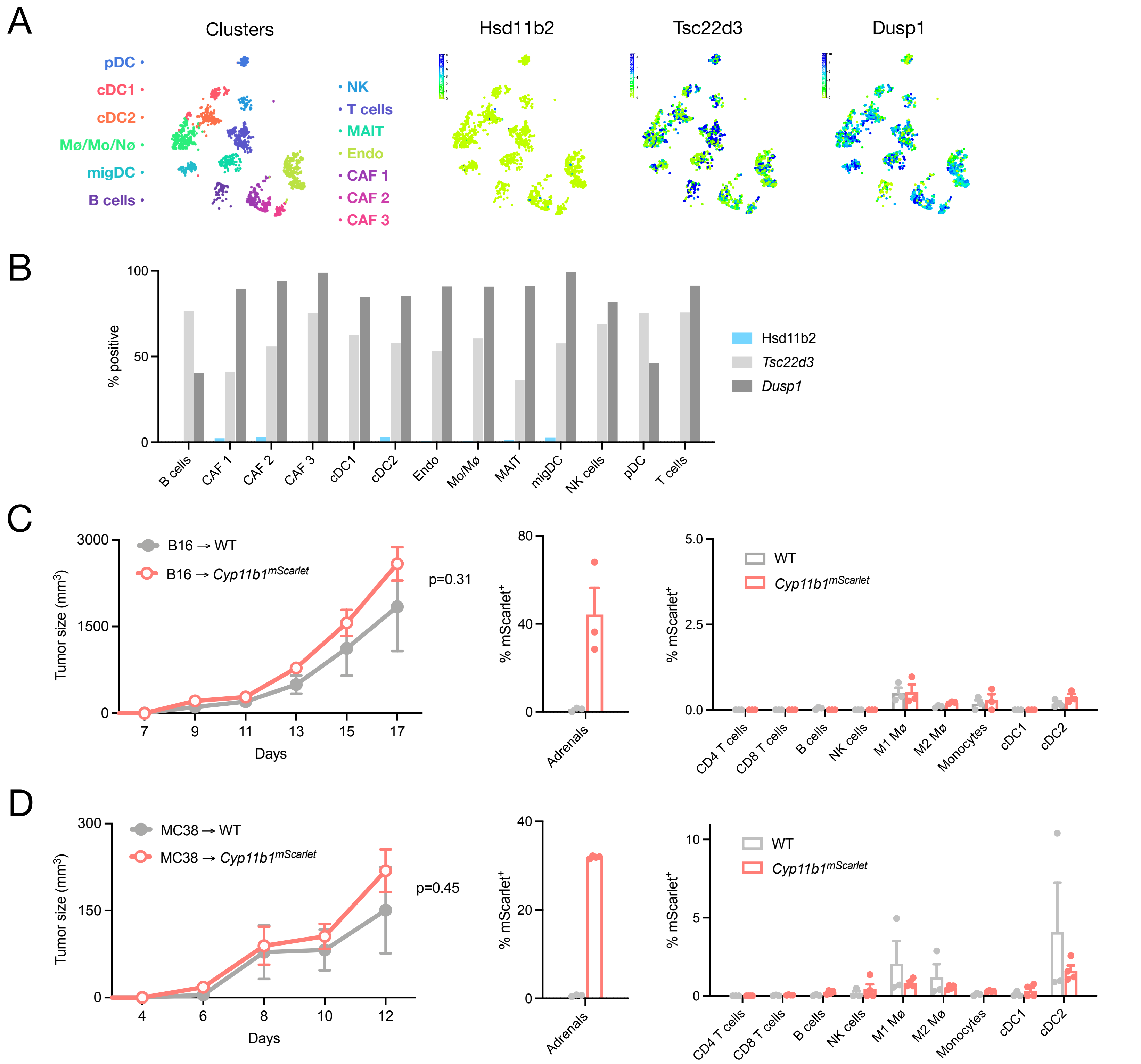

FIGURE S3 (related to Figure 3). Lack of *Cyp11b1* expression and activity in tumor-infiltrating cells.

(**A**) tSNE visualization of single-cell RNA profiles from mouse melanoma tumor-infiltrating cells. Gene expression data were obtained from the Wellcome Sanger Institute mouse genomes project and visualized using the cellxgene platform.
(**B**) Single-cell RNA from (A) showing the percent of cells in each cluster expressing glucocorticoid metabolic enzyme genes (left) and glucocorticoid response genes (right).
(**C, D**) Tumor growth of B16 (**B**) or MC38 (**C**) cells implanted into wild-type or *Cyp11b1*^{mScarlet} mice. The frequency of mScarlet^{+} in adrenal and tumor-infiltrating cells is shown at center and right, respectively. Data are presented as mean ± SEM.

FIGURE S4

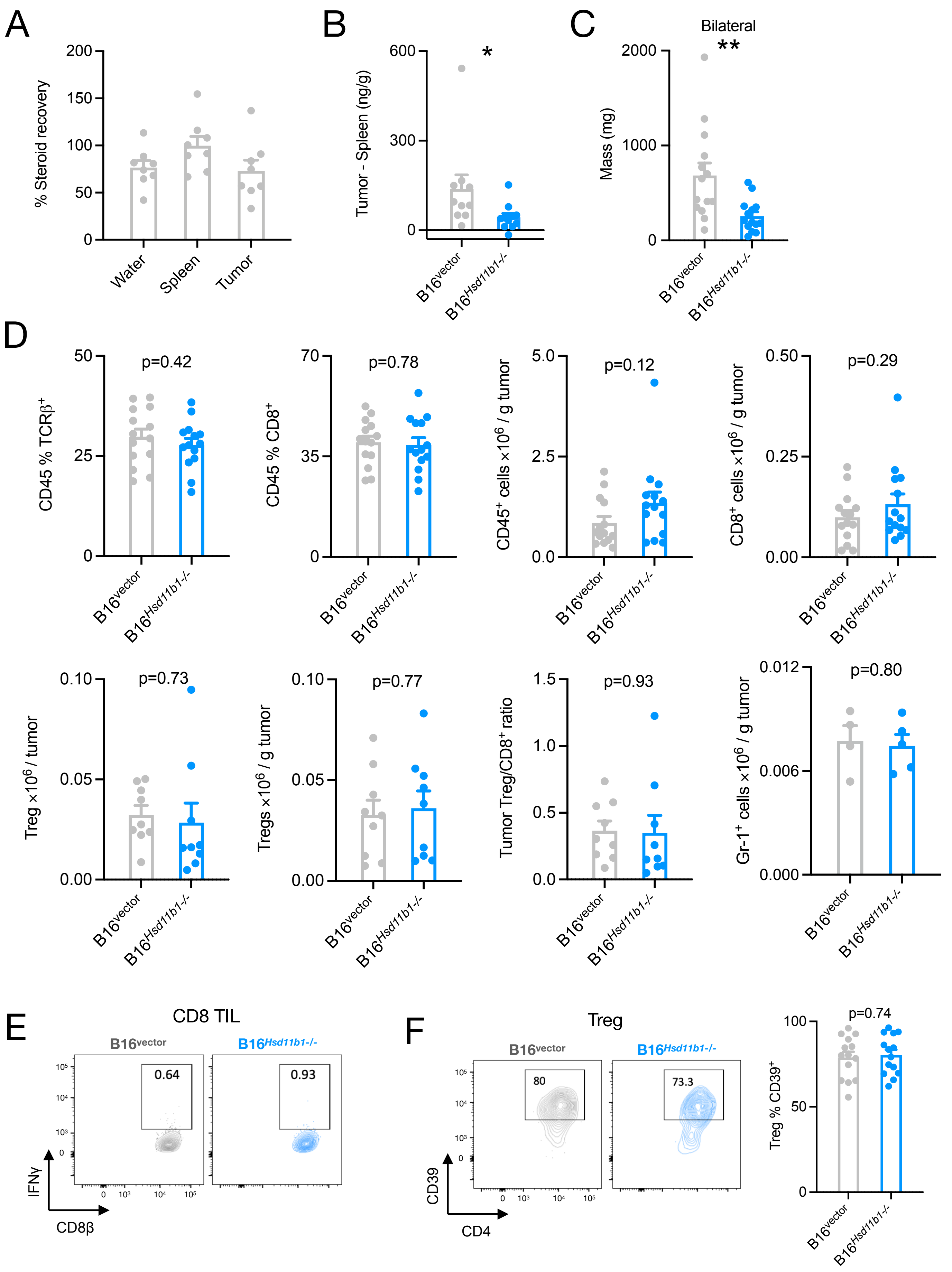

FIGURE S4 (related to Figure 4). Tumor-generated glucocorticoids inhibit infiltrating CD8 and CD4 T cell activity.

(**A**) Corticosterone recovery from water blanks, spleen tissue, and tumor tissue pools after solid phase extraction. Tissue pools were spiked with known amounts of corticosterone or vehicle, steroids extracted with C_{18} columns, and ELISA quantification of corticosterone used to determine corticosterone recovery values. Obtained tissue values were corrected for recovery.
(**B**) Difference in corticosterone concentration between B16 control or *Hsdd11b1*-/- tumor tissue and spleen tissue from the same mice.
(**C**) B16 control and *Hsd11b1*-/- tumor masses after bilateral implantation of both cell types into single recipient mice.
(**D**) Quantification of tumor-infiltrating cells in B16 control and *Hsd11b1*-/- tumors. Data are pooled from multiple experiments.
(**E**) Cytokine staining of unstimulated control tumor-infiltrating CD8 T cells.
(**F**) CD39 expression on Foxp3+ Treg cells from B16 control or *Hsd11b1*-/- tumors.
Data are presented as mean ± SEM and significance of p<0.10, <0.05, <0.01, <0.001 indicated as +, *, **, ***.

FIGURE S5

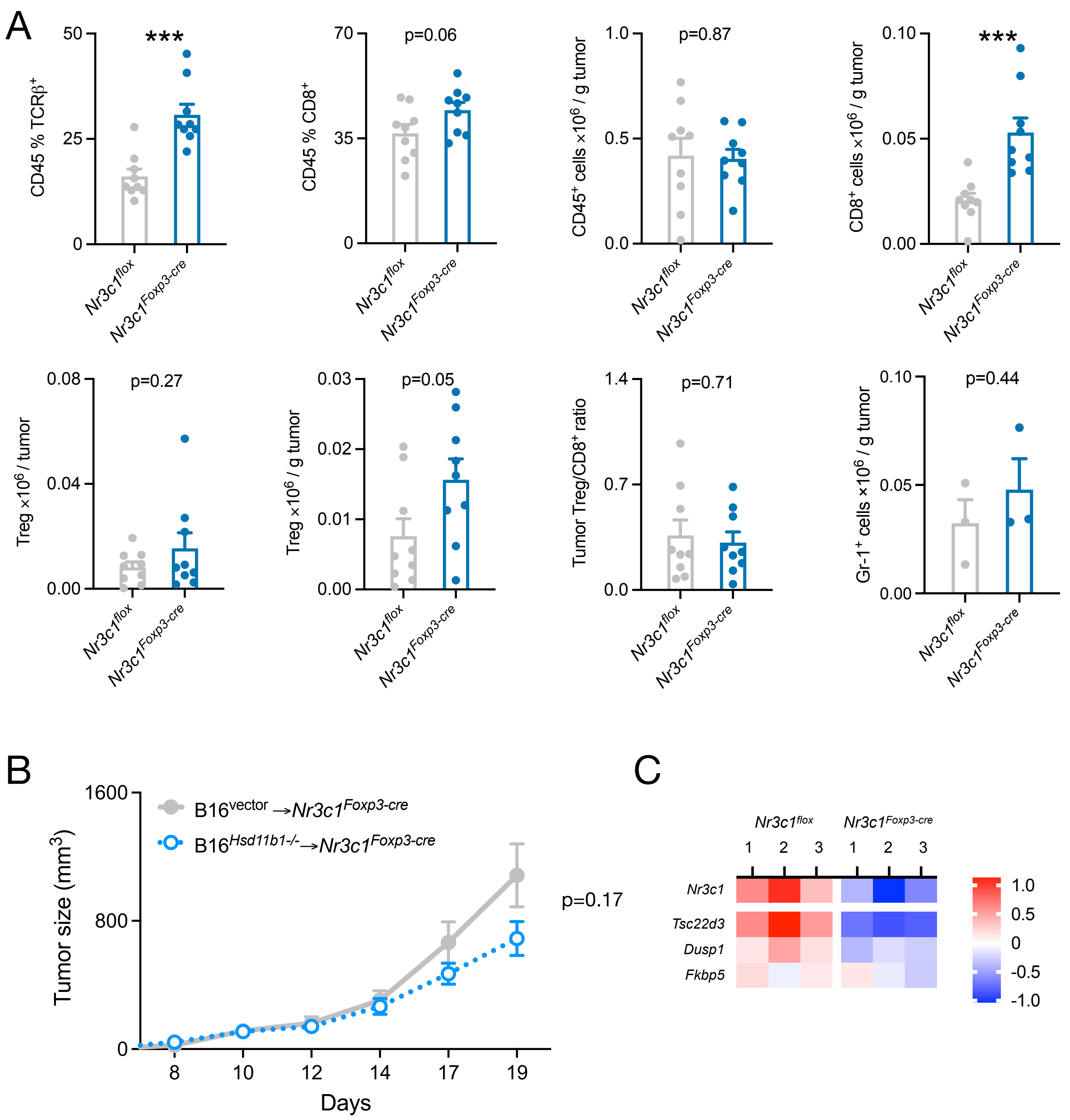

FIGURE S5 (related to Figure 5). Glucocorticoids promote tumor Treg activity.

(**A**) Quantification of tumor-infiltrating cells in B16 tumors implanted into control and *Nr3c1*$^{Foxp3\text{-}cre}$ mice. Data are pooled from multiple experiments, presented as mean ± SEM, and significance of p<0.05, <0.01, <0.001 indicated as *, **, ***.
(**B**) B16 control and Hsd11b1$^{-/-}$ cell tumor growth in *Nr3c1*$^{Foxp3\text{-}cre}$ mice (N=17,16), Pooled from 2 experiments.
(**C**) RNA-seq analysis *Nr3c1* and glucocorticoid-responsive genes in control and *Nr3c1*$^{Foxp3\text{-}cre}$ tumor-infiltrating Treg. Relative expression is shown as $\log_2$ fold change from the mean expression of all samples.

FIGURE S6

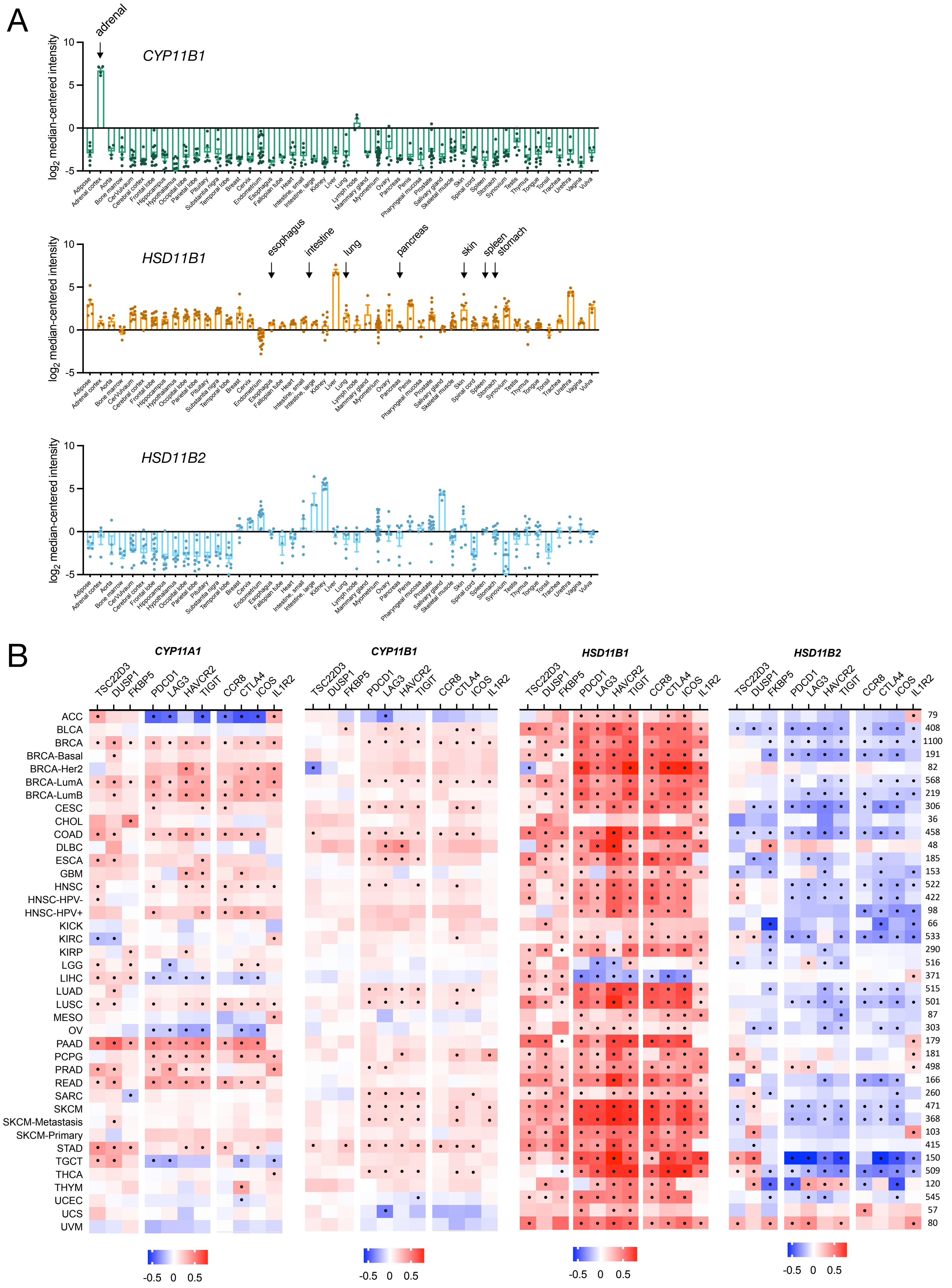

FIGURE S6 (related to Figure 6). *HSD11B1* is expressed across human tissues and tumor expression correlates with signature T cell exhaustion and immunosuppression genes.

(**A**) Relative gene expression of CYP11A1, CYP11B1, and HSD11B1 in healthy adult human tissues. Data were acquired on Oncomine.
(**B**) Correlation between expression of *CYP11A1*, *CYP11B1*, *HSD11B1*, or *HSD11B2* and glucocorticoid response genes (*TSC22D3*, *DUSP1*, *FKBP5*), T cell dysfunction genes (*PDCD1*, *LAG3*, *HAVCR2*, *TIGIT*), and Treg marker genes (*CCR8*, *CTLA4*, *ICOS*, *IL1R2*) in human cancers. TCGA gene expression data were analyzed using TIMER2.0 and are shown as heatmaps showing the strength of the correlation (partial spearman's rho) with significant correlations indicated with a black circle. Sample sizes are shown at far right.

FIGURE S7

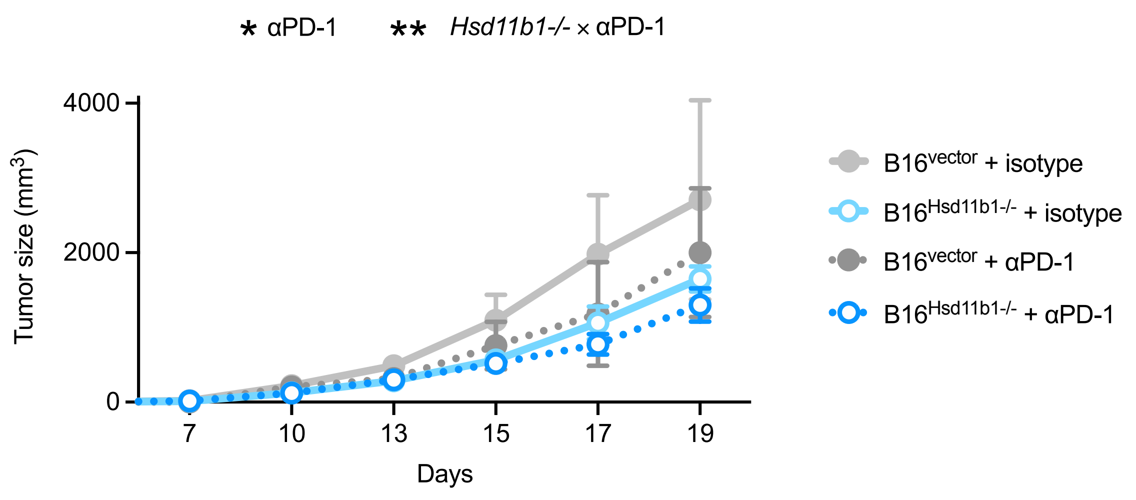

FIGURE S7 (related to Figure 7). ***Checkpoint blockade and Hsd11b1 deficiency have additive effects on tumor growth.***

B16 control and Hsd11b1$^{-/-}$ tumor growth in wild-type mice, treated with 10 mg/kg anti-PD-1 or rat IgG_{2a} isotype control (200 µL intraperitoneal) (N=4 per group). Treatments were administered on days 10, 12, and 14.
